# The liver clock modulates circadian rhythms in white adipose tissue

**DOI:** 10.1016/j.molmet.2025.102249

**Published:** 2025-09-12

**Authors:** Ivan Vlassakev, Christina Savva, Gianluca Renzi, Hema S. Ilamathi, Doste R. Mamand, Jacob G. Smith, Carolina M. Greco, Christopher Litwin, Qing Zhang, Leandro Velez, Angela Ma, Martin O. Bergo, Oscar P.B. Wiklander, Pura Muñoz-Cánovez, Niklas Mejhert, Marcus Seldin, Johan L.M. Björkegren, Paolo Sassone-Corsi, Kevin B. Koronowski, Salvador Aznar Benitah, Paul Petrus

**Affiliations:** 1 Department of Physiology and Pharmacology, C3, Karolinska Institutet, 171 77 Stockholm, Sweden; 2 Department of Medicine (H7), Karolinska Institutet, Stockholm 141 86, Sweden; 3 Department of Laboratory Medicine, Karolinska Institutet, 141 86 Stockholm, Sweden; 4 Breast Center, Karolinska Comprehensive Cancer Center, Karolinska University Hospital, Stockholm, Sweden; 5 Department of Cell Biology, Physiology and Immunology, School of Biology, University of Barcelona, Barcelona, Spain; 6 Institute of Biomedicine of the University of Barcelona (IBUB), University of Barcelona, Barcelona, Spain; 7 Department of Biomedical Sciences, Humanitas University, Via Rita Levi Montalcini 4 Pieve Emanuele, 20072, Milan, Italy; 8 IRCCS Humanitas Research Hospital, via Manzoni 56 Rozzano, 20089, Milan, Italy; 9 Department of Biochemistry and Structural Biology, University of Texas Health San Antonio, San Antonio, TX 78229, USA; 10 Department of Biological Chemistry, University of California, Irvine, USA; 11 Department of Genetics & Genomic Sciences, Institute of Genomics and Multiscale Biology, Icahn School of Medicine at Mount Sinai, New York, NY, 10029-6574, USA; 12 Department of Biosciences and Nutrition, H2, Karolinska Institutet, Stockholm 141 86, Sweden; 13 Department of Medical and Life Sciences (MELIS), Pompeu Fabra University (UPF), Parc de Recerca Biomèdica de Barcelona (PRBB), 08003 Barcelona, Spain; 14 Altos Labs, Inc., San Diego Institute of Science, San Diego, CA 92121, USA; 15 Center for Epigenetics and Metabolism, U1233 INSERM, Department of Biological Chemistry, University of California, Irvine, Irvine, CA 92697, USA; 16 Institute for Research in Biomedicine (IRB Barcelona), The Barcelona Institute of Science and Technology (BIST), 08028 Barcelona, Spain

**Keywords:** Circadian rhythms, Cross-tissue communication, Lipid metabolism, Cardiometabolic disease

## Abstract

Circadian rhythms are integral to maintaining metabolic health by temporally coordinating physiology across tissues. However, the mechanisms underlying circadian cross-tissue coordination remain poorly understood. In this study, we uncover a central role for the liver clock in regulating circadian rhythms in white adipose tissue (WAT). Using a hepatocyte-specific *Bmal1* knockout mouse model, we show that hepatic circadian control modulates lipid metabolism in WAT. In addition, by utilizing a model where functional clocks are restricted to the hepatocytes, we demonstrate that the liver clock alone integrates feeding cues to modulate circadian gene expression in WAT, including *Cebpa*, a key regulator of adipogenesis. We show that the hepatocyte clock regulates adipocyte *Cebpa* rhythmicity through secreted proteins. Further investigation identified one of the contributing mediators to be the adaptor protein 14-3-3η (*Ywhah*). The clinical relevance of the liver clock for systemic metabolic function is supported by human cohort data, which revealed a gene regulatory network, consisting of several clock-controlled liver genes, linked to cardiometabolic risk. These findings provide evidence for how the hepatocyte clock coordinates WAT physiology and highlights the core clock system as a potential therapeutic target to improve cardiometabolic health.

## Highlights

• The hepatic clock modulates transcriptional rhythms of lipid metabolism in white adipose tissue.
• The hepatocyte clock communicates with differentiating adipocytes through secreted proteins.
• The adaptor protein 14-3-3 η (*Ywhah*) contributes to circadian liver-adipose communication.
• The liver clock is a key driver of a gene regulatory network linked to cardiometabolic health.

## Graphical abstract

**Figure:**
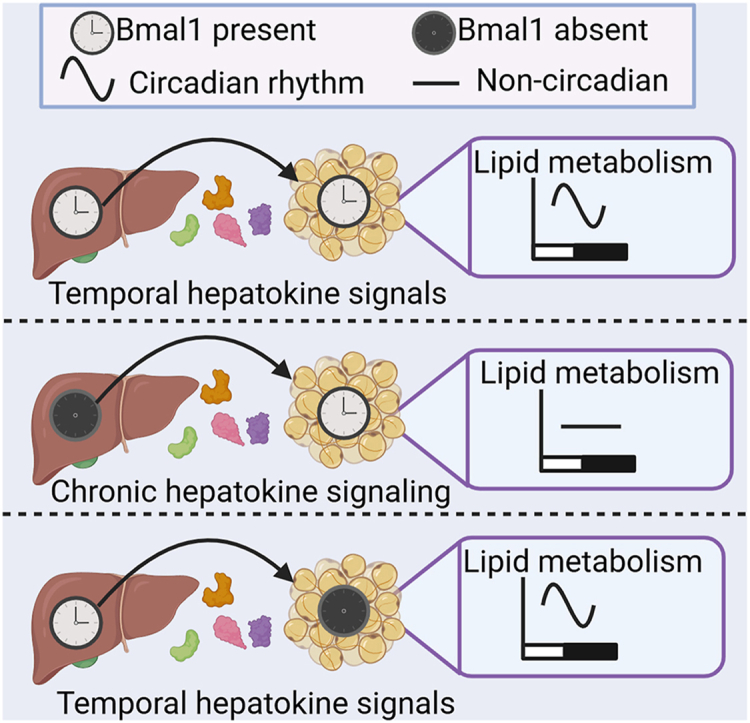

## 1 Introduction

Mammals, like most living organisms, possess genetically encoded molecular clocks that facilitate physiological functions aligned with the time of day. The clock operates through a transcriptional-translational feedback loop, where core clock genes (with the essential component *Bmal1*) and their protein products, regulate their own expression in a precisely controlled cycle over approximately 24 h [1]. Disruption of these processes is linked to an increased risk of diseases, including cardiometabolic disorders [2,3]. Hence, a better understanding of how the circadian system operates could reveal novel approaches to diagnosing and treating metabolic morbidities.

Circadian rhythms are synchronized by external cues (*Zeitgebers*), with light being the dominant one. Light entrains the clock in the suprachiasmatic nucleus (SCN) that in turn relays this information to control organism-wide circadian homeostasis [4,5]. Notably, clocks in peripheral organs, like the liver, can be uncoupled from the central pacemaker of the SCN by the *Zeitgeber* function of nutritional cues [6]. Much focus has been given to dissect the organization of tissue-specific clocks in controlling systems-wide circadian homeostasis [7,8].

We have begun to dissect the organization and communication between tissue clocks, and how this contributes to physiological regulation. Our recent findings suggest that the brain clock plays a crucial role in driving circadian rhythms in the periphery [[9], [10], [11]]. Yet, we have also showed that peripheral clocks are required to integrate, fine-tune and relay these signals (such as feed-fasting rhythms) for several physiological processes including muscle function, cell cycle and glucose homeostasis [[10], [11], [12], [13]]. Thus, defining the architecture of the circadian system may provide insights into its mechanistic links to metabolic homeostasis. Substantial evidence points to the role of hepatokines (for example FGF21, RBP4 and ANGPTL4) in sustaining systemic metabolic homeostasis by signalling to other metabolic organs, including the white adipose tissue (WAT) [14]. In fact, a recent study demonstrated that the circadian clock component REV-ERBα supresses hepatokines that stimulate systemic catabolism, including adipocyte lipolysis [15]. Hence, the hepatocyte clock may play a central role in organizing systemic circadian homeostasis by communicating with WAT. Accordingly, we hypothesized that the hepatocyte clock integrates feed-fasting cues and relays circadian signals to regulate WAT oscillations. By combining transgenic mouse models, innovative *in vitro* systems to study circadian inter-tissue communication, and supportive evidence from human clinical cohorts, we demonstrate that the hepatocyte clock is both necessary and sufficient to control WAT circadian rhythmicity by integrating feed-fasting cues. This provides a mechanistic framework highlighting the relevance of the hepatic clock in cardiometabolic health.

## 2 Results and discussion

### 2.1 The hepatocyte clock is required for rhythmic expression of lipid metabolic genes in white adipose tissue

**Figure 1:**
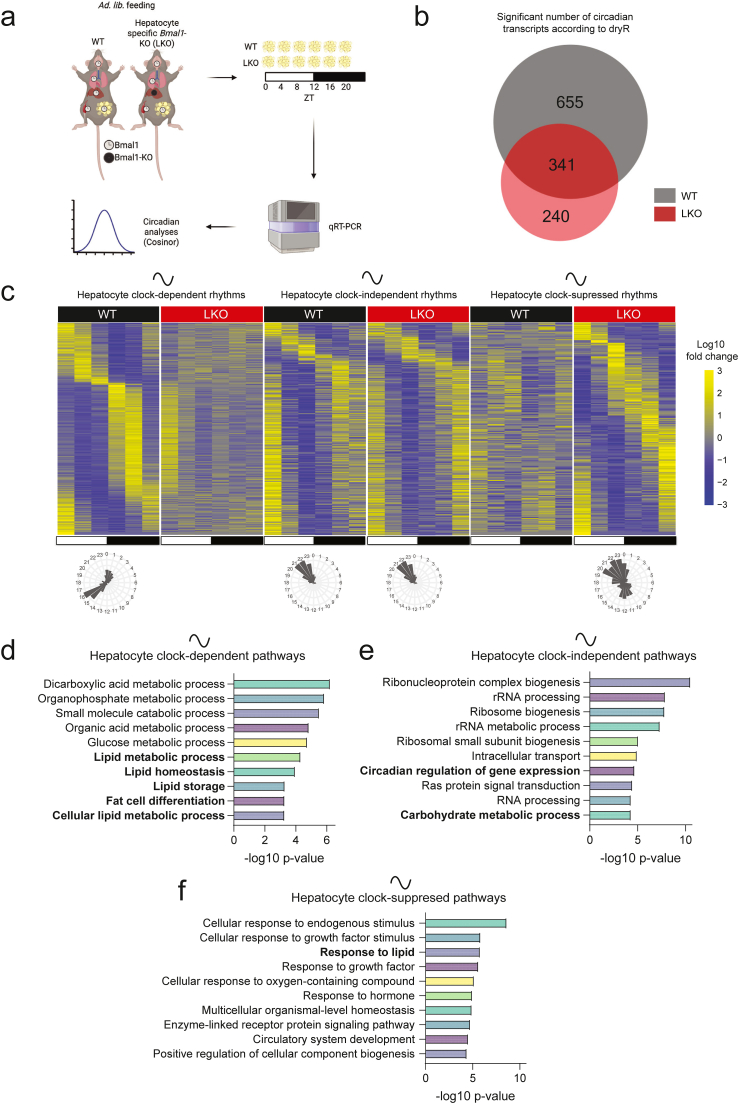
**Hepatocyte-specific *Bmal1* deletion modulates the circadian rhythmicity in white adipose tissue**. A) Schematic of the experimental design (Created with BioRender.com). Five biological replicates per genotype were sampled at each time point across the circadian cycle. B) Venn diagram showing the unique and shared significantly rhythmic transcripts according to dryR analyses in WAT between wild-type (WT) and hepatocyte-specific *Bmal1* knockout (LKO) mice. C) Phase-sorted heatmaps of WAT transcripts rhythmic in: WT only (hepatocyte clock-dependent), both genotypes (hepatocyte clock-independent), or LKO only (hepatocyte clocksuppressed). The radar charts illustrate the number of rhythmic transcripts with different peak phases within the groups. D‒F) Gene ontology enrichment of circadian transcripts identified in (C), showing the most significantly enriched pathways for each gene set. Pathways of interest are highlighted in bold.

To determine whether transcriptional rhythms in WAT depend on the hepatocyte clock, we used a hepatocyte-specific *Bmal1* knockout model (Figure 1A), in which the essential clock protein BMAL1 is absent specifically in the hepatocytes (Fig. S1a). The term LKO will be used to refer to these mice; an abbreviation for liver knock out. Of note, these mice have previously shown to display feeding rhythms comparative to those in wild-type (WT) mice under *Ad libitum* conditions [16]. To rule out feeding behavior and systemic metabolic parameters as potential confounders influencing WAT oscillations, we assessed a range of metabolic readouts, including body weight, fat pad mass (normalized to body weight), food intake, locomotor activity, respiratory exchange ratio (RER), energy expenditure (EE), and glucose tolerance in WT and LKO littermates (Fig. S1b-l). We found no significant differences between the groups in any of the parameters assessed, apart from glucose tolerance: LKO mice exhibited lower blood glucose levels during the first hour after the gavage (Fig. S1k-l), consistent with previous reports [17]. Despite the enhanced glucose clearance observed in LKO mice, the overall metabolic similarity between genotypes supports the use of this model to investigate the role of the hepatocyte clock in modulating WAT oscillations, with minor influence of other systemic metabolic confounders. To this end, we collected epididymal WAT samples from these mice at six *Zeitgeber* time (ZT) points and performed RNA-sequencing analyses to identify circadian transcriptional regulation influenced by the hepatocyte clock.

We first investigated the contribution of hepatocyte BMAL1 in coordinating WAT molecular clock rhythms. We found that rhythms of the core clock machinery in WAT were altered in LKO in comparison to WT (Fig. S1m). More specifically, the expression of *Bmal1* displayed a dampened amplitude in WAT of LKO mice compared to WT with significantly lower expression at ZT 0 and 20 while *Nr1d1* and *Dbp* increased in amplitude with significantly higher expression levels at ZT 12 and 8, respectively (Fig. S1m). *Per2* gained significant circadian rhythms in the absence of the hepatocyte clock (Fig. S1m). These results indicate that the core clock in WAT is modulated by the hepatocyte clock. These observations were not reported in a previous study investigating white adipose tissue from hepatocyte-specific *Bmal1* knockout mice [16]. To assess this further, we examined our RNA-sequencing dataset from WAT of LKO mice, which corroborated their findings; namely, no detectable differences in the rhythmic parameters of core clock gene expression (Supplemental table S1). The discrepancy in gene expression is potentially due to averaging effects across isoforms or limited sensitivity to low-expression transcripts. Thus, this prompted us to measure the protein levels of BMAL1 in WAT from WT and LKO mice across the 24-hour cycle. Western blot analyses suggested that WAT BMAL1 protein levels lost circadian rhythmicity in the absence of the hepatocyte clock (Fig. S1n), suggesting interconnectivity between tissue-specific clocks. However, these findings warrant further validation using complementary approaches in future studies.

Next, we explored the dependency of the hepatic-clock in regulating rhythms of global WAT transcription. Our results show that the hepatocyte clock is required to control the rhythmicity of 655 transcripts and supress the rhythms of 240 transcripts in the WAT (Figure 1B). Of note, only approximately one third (341/991 transcripts) of the rhythmic genes in WAT from WT mice oscillate independently of the hepatocyte clock.

Furthermore, only ten of these 341 transcripts displayed anti-phasic oscillations and 18 showed differences in mesor (mean expression level) between groups, suggesting that most hepatocyte clock-independent rhythms are conserved in their circadian parameters (Supplementary table 1). Most hepatocyte clock-dependent transcripts peaked at ZT 16, while the hepatocyte clock-independent and -supressed transcripts peaked at ZT 20-to-0 (Figure 1C). To understand which pathways in WAT are controlled by the hepatocyte clock, we next performed pathway analyses. These revealed that hepatocyte clock-dependent rhythms in WAT were enriched for lipid metabolic processes (Figure 1D). Conversely, carbohydrate metabolic processes kept their oscillations upon hepatocyte clock knock out (Figure 1E). Furthermore, deletion of the hepatocyte clock induced oscillations in genes enriched for response to lipids (Figure 1F), further highlighting the importance of the hepatocyte clock in modulating WAT lipid metabolism. Collectively, these findings suggest that the hepatocyte clock tunes the WAT clock and rewires the transcriptome, regulating rhythmicity of lipid metabolic genes.

### 2.2 The hepatocyte clock integrates feed-fasting cues to modulate transcriptional rhythms in white adipose tissue

**Figure 2:**
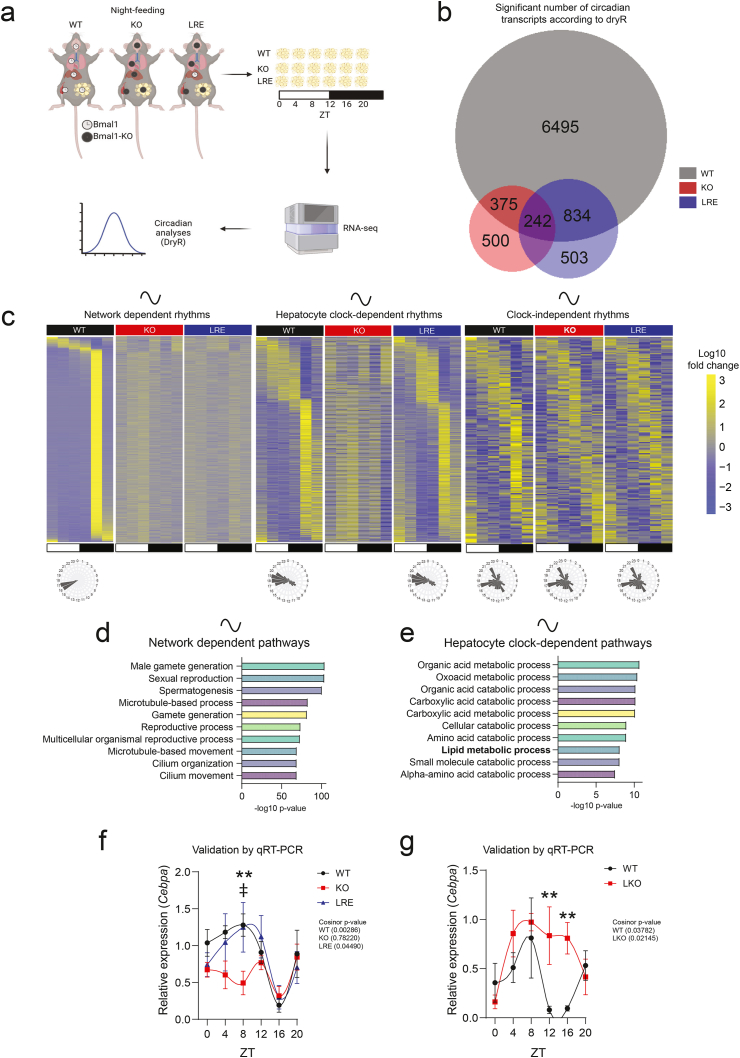
**The liver clock is sufficient to integrate feed-fasting cues to modulate transcriptional rhythms in white adipose tissue**. A) Schematic of the experimental design (Created with BioRender.com). Four biological replicates per genotype (wildtype [WT], full-body *Bmal1*-KO [KO], and hepatocyte clock reconstitution [LRE]) were sampled across circadian time. B) Venn diagram showing the unique and shared significantly rhythmic transcripts according to dryR analyses in WAT between WT, KO and LRE mice. C) Phase-sorted heatmaps and corresponding radar plots of rhythmic WAT genes categorized as: network-dependent (WT-specific), hepatocyte clockdependent (shared by WT and LRE), and clock-independent (common to all genotypes). The radar charts illustrate the number of rhythmic transcripts with different peak phases within the groups. D-E) Gene ontology enrichment of rhythmic transcripts identified in (C), showing significantly enriched pathways for each gene set. Pathways of interest are highlighted in bold. F) qPCR analysis of *Cebpa* expression in WAT from WT (black), KO (red) and LRE (blue) mice (n = 24 per genotype; four biological replicates per time point). Graphs represent mean values of the relative gene expression and error bars represent standard error of the mean. Cosinor *p*-values are shown; statistical comparisons between groups at individual time points were performed using two-way ANOVA with Fisher's LSD test. ∗Represents significant differences in the comparison between WT and KO and ‡ represents significance in the comparison between KO and LRE. ∗∗ p-value <0.01, ‡ < 0.05. g) qPCR analysis of *Cebpa* expression in WAT from WT (black) and LKO (red) mice (n = 30 per genotype; five biological replicates per time point). Graphs represent mean values of the relative gene expression and error bars represent standard error of the mean. Cosinor *p*-values are shown; statistical comparisons between groups at individual time points were performed using two-way ANOVA with Fisher's LSD test. ∗∗ p-value <0.01.

In the LKO model we found that the rhythmicity of the local WAT clock is tuned by the hepatocyte clock. Therefore, it is possible that global changes in WAT transcriptional rhythms, induced by the absence of the hepatocyte clock, could be mediated via the local WAT clock. To test for the sufficiency of the hepatic clock in integrating feeding cues to modulate WAT oscillations, we interrogated a previously described hepatocyte clock reconstituted (LRE) mouse model [18] in which the essential clock gene *Bmal1* is depleted in all tissues except for the hepatocytes. As controls, we conducted parallel studies in WT and full-body *Bmal1* knockout (KO) mice (Figure 2A). Importantly, under *ad libitum* feeding conditions, the LRE and KO mice display arrhythmic eating behaviours [13,18]. Therefore, for all these models, the mice had access to food only during the active phase (ZT12-0), in which they consumed similar amount of food, for two weeks to control for the lack of feed-fasting rhythms in LRE and KO mice [13]. Of note, the night-feeding regimen restored rhythmicity of EE and RER, albeit with differences in amplitude in relation to WT mice. However, no differences were observed between the LRE and KO mice, indicating that feeding-induced metabolic rhythms are unlikely to confound the differences in WAT oscillations between these groups [13]. Therefore, the night-fed LRE model was considered suitable for testing the sufficiency of the hepatic clock in integrating and subverting feed-fasting cues to control WAT oscillations. Thus, as for the LKO model, epididymal WAT was collected at six circadian time points for circadian analyses of transcription. Based on these samples, we confirmed that the core clock machinery in WAT was rhythmic in WT mice and nonfunctional in KO and LRE mice due to the absence of *Bmal1* in WAT (Fig. S2a). Hence, allowing us to investigate the role of the hepatic clock regulating transcriptional rhythms independent of the local clock in WAT. To interrogate the circadian liver-WAT communication, we next generated WAT transcriptomic data based on all samples collected and performed analyses to detect oscillating transcripts [19] (Supplementary table 2; methods). The sufficiency of the hepatocyte clock in integrating feeding cues to regulate WAT transcriptional rhythms was supported by a recovery of 834 genes with a significant circadian rhythm in LRE mice that also were rhythmic in WT mice but not in the KOs (Figure 2B). Of these, 801 transcripts were phase-aligned between WT and LRE (Figure 2C and Supplemental table S2), and 641 showed similar mesor, indicating substantial overlap in both timing and expression level (Supplemental table S2). Most of the rhythmic genes peaked in the middle of the active/fed state (ZT16, 4 h after lights off and with food access) (Figure 2C), which could be a result of the restricted eating window and is in line with previous studies [20]. Thus, the hepatocyte clock is sufficient to integrate feed-fasting cues and modulate a proportion of the circadian transcriptome in WAT. The 242 clock-independent oscillating transcripts (rhythmic in all genotypes) exhibited a broader distribution of peak phases, with 162 being phase-aligned and 155 showing similar mesor across genotypes (Figure 2C and Supplemental table S2).

To gain further insight into which pathways in WAT that are controlled by the hepatocyte clock, we performed pathway analyses of the network-dependent (requiring at least one other tissue clock), hepatocyte clock-dependent (restored rhythmicity by reconstituting the hepatocyte clock) and clock independent (rhythmic in the presence and absence of tissue clocks) (Figure 2C). The network-dependent transcripts were most strongly enriched for genes involved in reproductive processes (Figure 2D). This unexpected observation suggests a potential non-canonical role for these transcripts in adipose tissue physiology, as supported by a previous study [21]. To investigate whether this phenomenon is specific to murine WAT, we analysed the expression of representative transcripts identified in our dataset (Fig. S2b) using a publicly available portal containing human WAT single-cell RNA-sequencing data [22]. Interestingly, several spermatogenesis-associated genes (including *SPAGA* and *SPATA* family members) were expressed across multiple WAT cell types, including adipocytes (Fig. S2c), suggesting that these genes may have conserved, non-canonical functions in adipose tissue across species that warrant further investigation. Of note, the networkdependent rhythms did not significantly enrich for any pathway related to lipid metabolism. Intriguingly, pathway analyses of the hepatocyte clock dependent transcripts were enriched for lipid metabolic process as well as organic acid processes (Figure 2E), corroborating the observations in the LKO model. Importantly, transcripts enriched in lipid metabolic processes exhibited mesor differences between WT and LRE mice (Supplemental table S2), suggesting that while the hepatic clock integrates feeding cues to regulate oscillatory dynamics of this pathway in WAT, other tissue clocks may be required to establish and maintain baseline expression levels. Feedfasting rhythms *per se* (transcripts rhythmic in all genotypes) were insufficient to drive the rhythmicity of any pathway enrichments. However, when assessed individually, three key metabolic genes, *Adipor1*, *Apoe*, and *Ldlr*, fell into this category. These transcripts exhibited conserved phase, amplitude, and mesor across genotypes, although minor differences in expression levels were observed at ZT12 and ZT16 (Fig. S2d). These data highlight the essential role of the hepatocyte clock in regulating transcriptional rhythms of lipid metabolism in WAT.

A closer look into the hepatocyte-clock dependent rhythmic genes involved in lipid metabolic process identified *Cebpa*, encoding a central transcription factor regulating adipocyte differentiation (adipogenesis) [23]. Both the rhythmicity and expression level of *Cebpa* were restored in LRE mice while KO mice displayed loss of rhythms and significantly lower expression at ZT 8 (Figure 2F). To further investigate the dependency of WAT *Cebpa* expression on the hepatocyte clock, we went back to the LKO model.

The data suggest that *Cebpa* rhythms require the hepatocyte clock for the trough at ZT 12–16 (Figure 2G). To validate our findings and further assess the role of feeding rhythms in mediating *Cebpa* rhythmicity, we mined the dataset published by Manella et al. [16]. Consistent with our observations, *Cebpa* was rhythmic under *ad libitum* feeding but lost significant rhythmicity under a day-feeding regimen (food accessible only during the resting phase) (data not shown). Taken together, these findings suggest that the hepatocyte clock is both required and sufficient to integrate timely feed-fasting cues to modulate *Cebpa* rhythms in WAT.

### 2.3 In vitro characterization of circadian communication from hepatocytes to adipocytes

**Figure 3:**
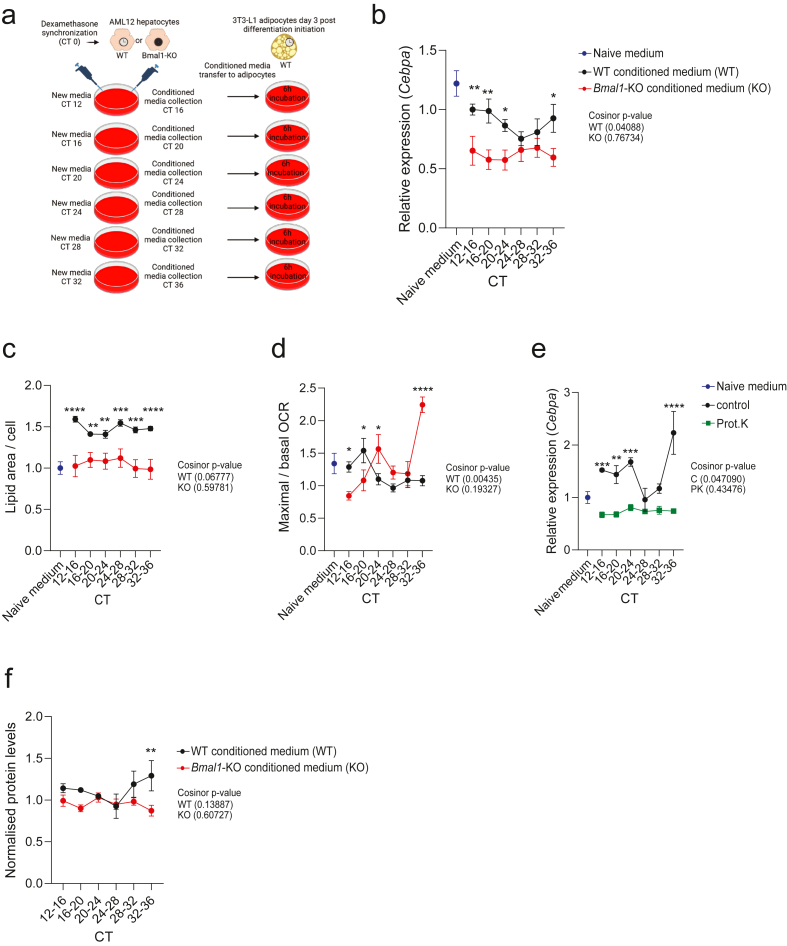
**Circadian hepatocyte-to-adipocyte communication is mediated through BMAL1-controlled hepatokines.** A) Schematic of the *in vitro* conditioned medium assay (Created with BioRender.com). The number of biological replicates is stated for each experiment in the respective panel. B) *Cebpa* expression in 3T3-L1 adipocytes treated for 6 h with naive medium (n = 8) or conditioned medium from AML12 WT or *Bmal1*-KO cells (n = 36 per group; six biological replicates per time point). Graphs represent mean values of the relative gene expression and error bars represent standard error of the mean. Cosinor *p*-values are shown; statistical comparisons between groups at individual time points were performed using two-way ANOVA with Fisher's LSD test. ∗p-value <0.05 ∗∗ p-value <0.01. C) Lipid accumulation in 3T3-L1 cells treated for 7 days with naive medium (n = 10) or conditioned medium from AML12 WT or *Bmal1*-KO cells (n = 54 per group, nine biological replicates per timepoint). Graphs represent mean values and error bars represent standard error of the mean. Cosinor *p*-values are shown; statistical comparisons between groups at individual time points were performed using two-way ANOVA with Fisher's LSD test. ∗∗ p-value <0.01, ∗∗∗p-value <0.001, ∗∗∗∗p-value <0.0001. D) Maximal over basal oxygen consumption rate of 3T3-L1 adipocytes treated for 6 h with naive medium (n = 8) or conditioned medium from AML12 WT or *Bmal1*-KO cells (n = 48, eight biological replicates per timepoint). Graphs represent mean values and error bars represent standard error of the mean. Cosinor *p*-values are shown; statistical comparisons between groups at individual time points were performed using two-way ANOVA with Fisher's LSD test. ∗p-value <0.05 ∗∗∗∗ p-value <0.0001. E) *Cebpa* expression in 3T3L1 adipocytes treated for 6 h with naive, EDTA-treated, or proteinase K–treated conditioned medium (n = 4 per time point per condition). Graphs represent mean values of the relative gene expression and error bars represent standard error of the mean. Cosinor *p*-values are shown; statistical comparisons between groups at individual time points were performed using two-way ANOVA with Fisher's LSD test. ∗∗ p-value <0.01, ∗∗∗p-value <0.001, ∗∗∗∗p-value <0.0001. F) Normalised levels of secreted proteins from conditioned medium from AML12 WT and *Bmal1*-KO cells (n = 36 per group, six biological replicates per timepoint). Graphs represent mean values and error bars represent standard error of the mean. Cosinor *p*-values are shown; statistical comparisons between groups at individual time points were performed using two-way ANOVA with Fisher's LSD test.

Circadian inter-organ communication involves endocrine, metabolic, and neuronal signaling [7,24], Thus, the circadian hepatic signals regulating WAT rhythms may involve both indirect- and direct-communication. To assess and manipulate the direct signals mediated by the hepatocyte clock to adipocytes at a critical point during differentiation, we developed a system studying cross-tissue circadian communication *in vitro*. For this, we first synchronized AML12 hepatocytes with a dexamethasone shock and thereafter collected conditioned media within specific time-windows. This media was subsequently transferred to unsynchronized, differentiating 3T3-L1 adipocytes (three days post induction of adipogenesis), and effects on transcription and cell function were assessed (Figure 3A). Naive media, not conditioned with AML12 cells prior to 3T3-L1 incubation, was used as a control. An early time point in adipocyte differentiation was selected for these experiments to capture effects on adipocytes around the onset for irreversible adipogenic commitment [25]. Parallel experiments were performed on AML12 cells, where *Bmal1* was knocked out using Cas9-mediated engineering which resulted in a significant downregulation in BMAL1 protein levels in the cell population (Fig. S3a) without influencing cell viability (Fig. S3b). Circadian rhythmicity of all clock genes measured was significant in synchronized WT AML12 cells and disrupted in *Bmal1*-KO cells (Fig. S3c). While AML12 conditioned media induced some cell death in 3T3-L1 cells compared to naive control media, no significant differences were observed between the experimental groups (Fig. S3d). Thus, cell viability was not considered a confounding factor in interpreting the role of cell-specific clocks in adipocyte function. Thus, this *in vitro* model was considered suitable to study the communication between the hepatocyte clock and differentiating adipocytes through secreted factors. First, we assessed the effect of the hepatocyte conditioned media on adipocyte *Cebpa* expression. Media conditioned with AML12 cells suppressed *Cebpa* expression in relation to naive media, irrespective of the AML12 cell genotype (Figure 3B). Media from synchronized WT AML12 cells controlled 3T3-L1 *Cebpa* expression in a temporal manner, resulting in a significant circadian rhythm (Figure 3B). This was not the case in 3T3-L1 cells treated with conditioned media taken from *Bmal1*-KO AML12 cells (Figure 3B). Thus, the direct circadian signals mediated by the hepatocyte clock, regulating *Cebpa*, could be captured with this experimental design. To assess whether these effects are independent of the local adipocyte clock, the treatment was repeated in 3T3-L1 *Bmal1*-KO cells (Fig. S3e).

Cas9-mediated engineering of 3T3-L1 cells led to a comparable downregulation of BMAL1 protein levels as observed in AML12 cells (Fig. S3f). In concordance with the *in vivo* models, hepatocyte signals regulate circadian *Cebpa* rhythms independent of the local adipocyte clock, albeit with a phase shift in comparison to adipocytes harbouring the clock (Fig. S3g). To further assess the role of BMAL1 in regulating *Cebpa* rhythms, we synchronized WT and *Bmal1*-KO 3T3-L1 cells. Synchronization induced significant circadian rhythmicity of *Bmal1* in WT, but not in KO cells (Fig. S3h), while *Cebpa* oscillations increased in amplitude in the absence of BMAL1 (Fig. S3i), indicating that intrinsic *Cebpa* rhythmicity is suppressed by the local adipocyte clock. Together, the results indicate that the hepatocyte- and the adipocyte clocks coordinate transcriptional rhythms of *Cebpa* in adipocytes.

### 2.4 The hepatocyte clock controls adipocyte lipid accumulation and respiration

We next aimed to investigate the role of the hepatocyte clock in regulating adipocyte physiology. Of note, LRE mice, subjected to night-time feeding, display similar respiration, body weights [13] and glucose tolerance [12] compared to KO mice. Hence, the systemic effects of the signalling from the hepatocyte-clock to WAT likely serve as a fine-tuning mechanism of the circadian system. These fine-tuning effects could, nevertheless, be captured in our *in vitro* model.

The differential regulation of genes involved in fatty acid metabolism led us to investigate the role of clock-driven hepatokines on adipocyte lipid accumulation and respiration. To this end, we incubated 3T3-L1 pre-adipocytes with conditioned media from the synchronized AML12 hepatocytes throughout the adipogenic differentiation process (daily media change over 7 days) and subsequently measured adipocyte lipid accumulation. Conditioned media from hepatocytes regulated rhythms (trend toward significant circadian rhythm) of lipid accumulation which were significantly higher in the group treated with WT conditioned media in comparison to adipocytes treated with media from *Bmal1*-KO hepatocytes (Figure 3C and S3j). The latter displayed lipid accumulation similar to cells treated with naive control media (Fig. 3C) These results support the notion that the hepatocyte clock mediates signals that promotes lipid accumulation in adipocytes.

We next assessed oxygen consumption in the 3T3-L1 cells treated with hepatocyte conditioned media for 6 h. The maximum over basal oxygen consumption ratio represents the spare respiratory capacity which, in our system, displayed circadian rhythmicity in adipocytes treated with WT conditioned media but not in adipocytes treated with conditioned media from *Bmal1*-KO hepatocytes (Figure 3D). Both genotypes displayed spare respiratory capacities overlapping with cells treated with naive control media (Figure 3D). The differences in the early time-points (CT 12–20) were a result of a lower basal- and higher maximum respiration in adipocytes treated with WT-compared to *Bmal1*-KO- conditioned media, while this relationship was inverted at CT 32–36 (Fig. S3k). Collectively, our findings highlight a role of the hepatocyte clock in contributing to the temporal regulation of adipocyte physiology.

### 2.5 Identification of clock-controlled hepatokines regulating adipocyte function

The direct circadian communication from the liver to WAT may be facilitated through metabolite and/or protein signalling. Lipid species are recognized as key regulators of WAT physiology [26]. However, our previous findings do not indicate that hepatocyte reconstitution of *Bmal1* rescues the oscillations of lipid metabolites in liver [13], rendering the focus towards proteins secreted from the liver. To validate the contribution of hepatokine proteins in regulating *Cebpa* rhythms, we treated conditioned media collected from AML12 cells with proteinase K to degrade proteins before transferring it to adipocytes. Control media, boiled with EDTA before treating 3T3-L1 cells, were used as a comparison. Proteinase K treatment reduced the protein levels in the media (Fig. S4a). Transcription of *Cebpa* in adipocytes treated with control media displayed significant circadian rhythmicity (Figure 3E). Conversely, the proteinase K treated group lost rhythmicity of *Cebpa* expression which was also significantly lower at CT 12–24 and CT 32–36 (Figure 3E). Of note, while WT conditioned media confirmed temporal regulation of *Cebpa*, we observed that boiling and EDTA treatment prior to transfer led to increased *Cebpa* expression compared to naive media, indicating that these media processing steps may influence cellular responses. Nevertheless, these results suggest a contribution from hepatokines in mediating *Cebpa* rhythmicity in adipocytes. We speculated whether the hepatocyte clock could modulate adipocyte function by regulating the rhythmicity of total protein secretion. To explore this, we precipitated proteins from AML12 cell-conditioned media, subjected them to SDSPAGE, and performed Ponceau staining (Fig. S4b). The results revealed a trend toward circadian rhythmicity of total protein secretion in WT but not *Bmal1*-KO cells, with significantly higher protein levels observed at CT32–36 (Figure 3F). These findings suggest that BMAL1 may contribute to the temporal regulation of overall protein secretion, although they do not preclude the involvement of specific hepatokines in mediating clock-driven signals.

**Figure 4:**
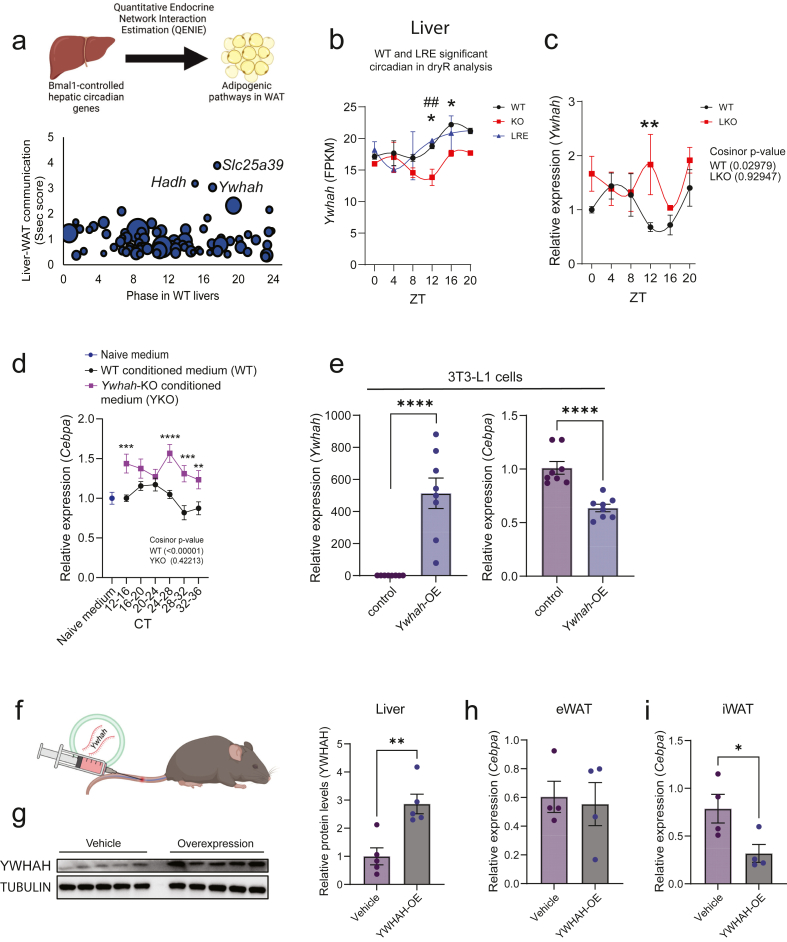
**The adaptor protein YWHAH contributes to circadian hepatocyte-toadipocyte communication.** A) Computational screen (Schematic representation created with BioRender.com) of liver-derived rhythmic genes predicted to affect adipocyte gene expression. Bubble size = amplitude of the gene in liver from LRE mice; X = ZT peak phase of the gene in livers from LRE mice; Y = score for the respective gene in liver predicting adipogenic gene expression in adipose tissue (Ssec-score). B) RNA-sequencing data showing the *Ywhah* expression in the livers from WT (black), KO (red) and LRE (blue) mice. Data is shown as Fragments Per Kilobase Million (FPKM). The graph represents mean values of FPKM, and error bars represent standard error of the mean. Statistical comparisons between groups at individual time points were performed using two-way ANOVA with Fisher's LSD test. ∗Represents significance in the comparison between WT and KO and # represents significance in the comparison between WT and LRE. ∗p-value <0.05, ##p-value <0.01. C) qPCR analysis of *Ywhah* expression in livers from WT (black) and LKO (red) mice (n = 24, four biological replicates per time point). The graph represents mean values of the relative gene expression and error bars represent standard error of the mean. Cosinor *p*-values are shown; statistical comparisons between groups at individual time points were performed using two-way ANOVA with Fisher's LSD test. ∗∗ p-value <0.01. D) *Cebpa* expression in 3T3-L1 cells treated with naive medium (n = 12) or conditioned medium from AML12 WT (n = 72) or *Ywhah*-KO cells (n = 69) for 6 h. Outliers were identified using the Robust regression and Outlier removal (ROUT). Number of biological replicates for each conditioned medium treatment is at least nine for each timepoint. Graphs represent mean values of the relative gene expression and error bars represent standard error of the mean. Cosinor *p*-values are shown; statistical comparisons between groups at individual time points were performed using two-way ANOVA with Fisher's LSD test. ∗∗ p-value <0.01, ∗∗∗p-value <0.001, ∗∗∗∗p-value <0.0001. E) *Ywhah* and *Cebpa* expression in 3T3-L1 cells overexpressing *Ywhah* vs controls (n = 8). A student unpaired t-test was performed to determine significant differences between groups. ∗∗∗∗ p-value <0000.1. F) Schematic of the tail vein injection of *Ywhah* mRNA in mice (Created with BioRender.com). G) Western blot of YWHAH and TUBULIN in livers from injected mice (n = 5 per group). A student unpaired t-test was performed to determine significant differences in protein levels. ∗∗ p-value <0.01. h) *Cebpa* expression in eWAT and iWAT from vehicle vs *Ywhah* mRNA-injected mice (n = 4 per group). Outliers were identified using the Robust regression and Outlier removal (ROUT). A student unpaired t-test was performed to determine significant differences between groups. ∗ p-value <0.05.

To identify candidate factors controlled by the hepatocyte clock that influence adipogenic transcription in WAT, particularly genes involved in fat cell lipid accumulation, we first defined rhythmic hepatic genes requiring BMAL1. To this end, we performed RNA-sequencing analyses of synchronized WT and *Bmal1*-KO AML12 cells (Supplementary table 3) and mined our published dataset [13] of the WT, KO, and LRE liver transcriptome that we re-analysed using DryR (Supplementary table 4; methods). Clock-driven hepatic transcripts (both *in vivo* and *in vitro*) were identified and then subjected to a bioinformatic tool predicting endocrine signalling, utilizing a genetically diverse mouse panel [27] (Figure 4A; methods). Among the 81 hepatocyteclock-driven genes, three stood out and displayed prediction scores (Ssec scores) greater than three (Figure 4A). Two of these top hits encode mitochondrial proteins (*Slc25a39* and *Hadh*), while the third, *Ywhah*, has been shown to be externalized in extracellular vesicles (EVs) [28]. This gene encodes 14-3-3η which belongs to a family of adaptor proteins involved in signal transduction, including insulin signalling [29]. Therefore, *Ywhah* in hepatocyte EVs could conceivably contribute to adipocyte rhythms by modulating various signalling cascades. We confirmed that hepatic transcriptional rhythmicity of *Ywhah* is controlled by the clock, both *in vivo* and *in vitro* (Figure 4B–C and Supplementary table S3). Hepatic levels of YWHAH protein were reduced in the absence of the hepatocyte clock, at ZT 8 but not 20, and restoration of the hepatocyte clock was sufficient to restore these levels (Fig S4c). These data were corroborated in the hepatocyte-specific *Bmal1* KO model (Fig S4d). The direct control of *Ywhah* by BMAL1 was further supported by our previously published ChIP-seq dataset from LRE mice [18]. We next assessed the presence of YWHAH in *ex vivo* collected EVs from livers. First, successful EV isolation was confirmed by the detection of established EV markers TSG101, CD63, and CD81 (Fig. S4e), as well as by Cryogenic Electron Microscopy (cryo-EM) (Fig. S4f) and Dynamic Light Scattering (DLS) (Fig. S4g). Thereafter, we verified the presence of YWHAH within these EVs (Fig. S4h). Furthermore, we confirmed that AML12 cells externalize EVs, albeit to a limited extent, as demonstrated by Western blot detection of TSG101 and CD63 (Fig. S4i), Transmission Electron Microscopy (TEM) (Fig S4j), and Nanoparticle Tracking Analysis (NTA) (Fig S4k). The presence of YWHAH in EVs was confirmed in our *in vitro* system using conditioned media from AML12 cells; however, YWHAH was more strongly enriched in the free protein fraction (Fig. S4l). Hence, our data suggest that YWHAH in the *in vitro* system does not reflect the *in vivo* regulation of its externalization, yet the effects of YWHAH on WAT can still be validated using this approach.

To test whether YWHAH is required for hepatocyte-to-adipocyte crosstalk, we employed Cas9 engineering to partially knock out the gene in a population of AML12 hepatocytes and repeated our *in vitro* intervention using these cells. The Cas9 mediated a 60% reduction of *Ywhah* expression in the cell population (Fig. S5a) without influencing cell viability (Fig. S5b). Treatment with the conditioned media from *Ywhah*-KO hepatocyte on 3T3-L1 adipocytes resulted in the loss of circadian rhythmicity in *Cebpa* expression (Figure 4D). As observed in previous experiments, media conditioned with AML12 cells induced some cell death compared to naive media; however, no significant differences were detected between the experimental groups (Fig. S5c). Furthermore, *Ywhah*-KO conditioned media treatment led to reduced lipid accumulation compared to WT conditioned media at all time points except CT24–32 (Fig. S5d), suggesting that additional clock-controlled hepatokines contribute to the regulation of adipocyte lipid storage during these circadian time points. In addition, *Ywhah*-KO conditioned media altered cellular respiration at CT12-20 compared to WT (Fig. S5e), further supporting the involvement of additional clock-controlled hepatokines in regulating adipocyte metabolic function. These results suggest that *Ywhah* is one of several clock-driven hepatokines regulating adipocyte functions. To test the direct effect of *Ywhah* on adipocyte *Cebpa* expression, we overexpressed *Ywhah* using the T7 ARCA mRNA technique by electroporating 3T3-L1 adipocytes at day 3 post differentiation initiation. In line with the observations made using the coculture system, *Cebpa* expression was repressed by local *Ywhah* overexpression (Figure 4E). To validate the *in vivo* relevance of these observations, we utilized the T7 ARCA mRNA method in a mouse model. The mRNA was encapsulated in lipid nanoparticles and administered via tail vein injection in 10-week-old C57BL/6 male mice at ZT8 (Figure 4F). This led to an approximately 3-fold increase in hepatic YWHAH protein levels (Figure 4G). However, this overexpression did not alter *Cebpa* expression in epididymal white adipose tissue (eWAT) (Figure 4H), suggesting that *Ywhah* is not sufficient to regulate *Cebpa* in this depot and that *Cebpa* rhythmicity in eWAT is likely controlled by other hepatocyte clock-dependent mechanisms.

This prompted us to question whether our findings were *in vitro* artifacts or if *Ywhah* could regulate *Cebpa* expression in a depot-specific manner. Indeed, *Cebpa* expression was significantly downregulated in inguinal WAT (iWAT) following *Ywhah* mRNA overexpression (Figure 4I). These findings support the validity of our approach to identify novel liver-to-WAT signalling pathways and highlight the importance of depot specific mechanisms, which warrant further investigation in future studies.

In sum, these data suggest that the hepatocyte clock controls adipocyte circadian rhythms of *Cebpa* through hepatokine signalling in a depot-specific manner.

### 2.6 Association between hepatic *YWHAH* expression and cardiometabolic health in humans

**Figure 5:**
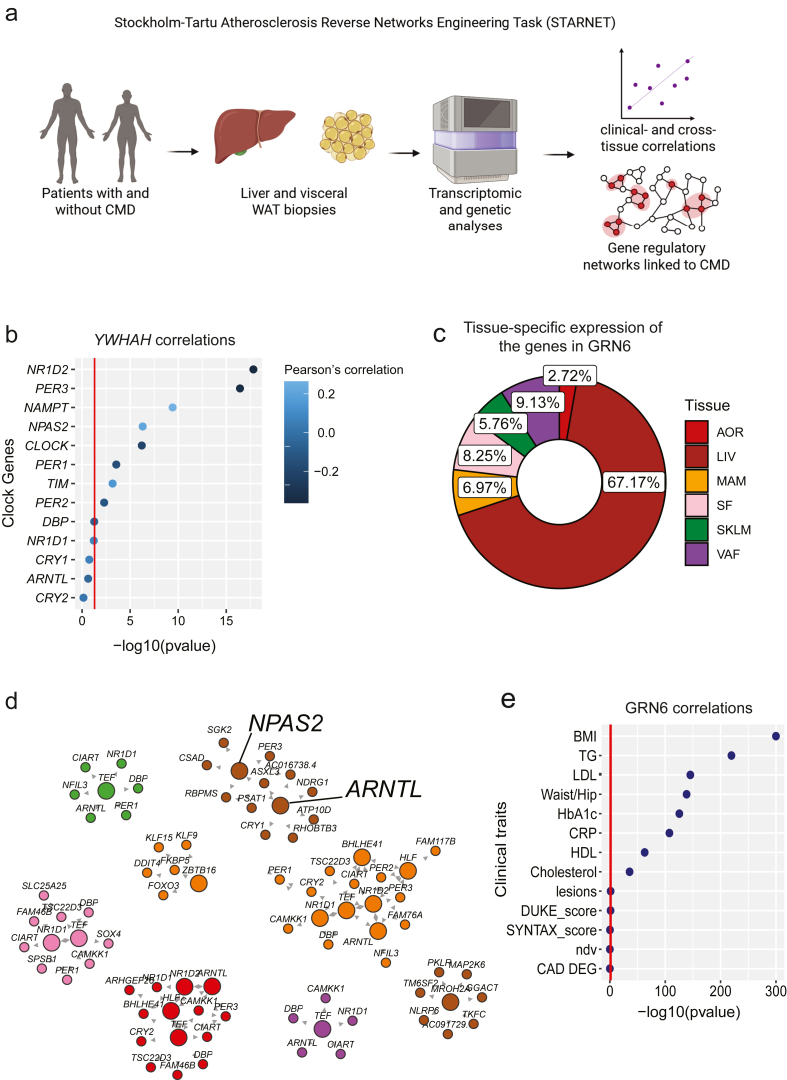
**Hepatic clock-controlled circadian genes are enriched in a gene regulatory network linked to cardiometabolic health**. A) Schematic representation of the clinical cohort STARNET (Created with BioRender.com). B) Age-, sex- and BMIadjusted Pearson correlations between hepatic *YWHAH* expression and core circadian clock genes. The red horizontal line denotes the significance threshold at –log10(pvalue). C) Pie chart illustrating the contribution of six organs to gene regulatory network 6 (GRN6). AOR: aortic wall; LIV: liver; MAM: mammary artery; SF: subcutaneous fat; SKLM: skeletal muscle; VAF: visceral fat. D) Network map showing predicted tissuespecific regulatory drivers and their nearest neighbours within GRN6. Node colours correspond to the tissues defined in (C). E) Correlations between GRN6 and clinical parameters relevant to cardiometabolic disease (body mass index [BMI], triglycerides [TG], low density lipoprotein [LDL], waist-to-hip ratio [Waist/Hip], glycosylated haemoglobin [HbA1c], c-reactive protein [CRP], high density lipoprotein [HDL], coronary artery disease complexity score [SYNTAX score], coronary artery disease severity score [DUKE], non-diseased vessels [ndv], coronary artery disease differentially expressed genes [CAD DEG]). Red line denotes statistical significance threshold (–log10(p-value)).

To understand the human relevance of *YWHAH*, we analysed liver and visceral WAT transcriptomic datasets from the well-characterized genetic-to-multi-omics Stockholm Tartu Atherosclerosis Reverse Networks Engineering Task (STARNET) study (Figure 5A) [30,31]. In brief, STARNET consists of nearly 2000 cardiometabolic disease (CMD) patients with and without coronary artery disease (CAD) from whom they sampled blood atherosclerotic and non-atherosclerotic arterial wall, liver, skeletal muscle as well as visceral abdominal (VAF) and subcutaneous fat (SF) during open-heart surgery. By exploring >500 RNA-sequencing profiles each from liver and VAF, we correlated *YWHAH* expression in the liver with genes in VAF. Pathway analyses of the significantly correlating transcripts in the VAF revealed positive association with immune response pathways and negative association with cholesterol- and lipid–metabolism pathways (Fig. S6a), with the latter supporting our findings in mice. Moreover, hepatic *YWHAH* expression correlated to several clinical parameters relevant for cardiometabolic disease, including systemic inflammation (C-reactive protein (CRP)) and blood lipids (Fig. S6b). However, these results are purely correlative and might be regulated by other factors associated with *YWHAH* expression. In fact, within the liver, several clock genes significantly correlated with *YWHAH* (Figure 5B), indicating that the correlations between *YWHAH* and cardiometabolic health might be driven by a larger gene regulatory network (GRN) consisting of several clock-controlled factors.

### 2.7 The core clock machinery is a key driver in a gene regulatory network strongly associated with cardiometabolic disease

The STARNET cohort has previously been used to identify 224 tissue-specific and cross-tissue GRNs forming mechanistic framework with high relevance for CMD [31].

STARNET applies probabilistic Bayesian network modelling based on genetics, gene expression and transcription factors to infer gene regulatory relationships facilitating assessment of dependencies within gene modules. Hence, an enrichment of genes associated with cardiometabolic health parameters within a GRN, indicates that these are causally linked to cardiometabolic health. Using this unique human resource, we investigated the enriched GRNs for the hepatocyte-clock driven transcriptional rhythms extracted from our murine transgenic mouse models (Supplementary table 4). This analysis identified GRN 6, consisting of primarily transcripts from the liver (Figure 5C and Supplemental table S5). This cross-tissue network included components of the core clock machinery as key drivers in multiple tissues, including the liver (Figure 5D), suggesting that the clock system itself is likely regulating cardiometabolic risk factors. Specifically, GRN 6 displayed a strong enrichment of genes associated with BMI, waistto-hip ratio, blood lipids and systemic inflammation (CRP) (Figure 5E). Among the clinical cardiometabolic parameters, CRP displayed a unique strong positive association to the key drivers (*NPAS2* and *ARNTL*, the latter encoding BMAL1) in the liver (Fig. S6c). These analyses, combined with our observations in the animal models, suggest that hepatic circadian rhythms contribute to systemic lipid homeostasis and inflammation. Our findings highlight the liver clock as a potential target for therapies treating cardiometabolic disease that warrants future experimental validation.

Previous research on the link between circadian rhythms and WAT homeostasis has primarily focused on the local clock in adipocytes and their progenitor cells [32]. However, recent discoveries suggest that the hepatocyte clock plays a role in buffering feeding cues to control transcriptional rhythms in other organs [16]. Conceptually, our study adds to the understanding of how the circadian network is organized by proposing a central role of the hepatocyte clock in modulating WAT transcriptional rhythms. Circadian communication from WAT, or other tissues, back to the liver likely plays a role and warrants further characterization in future studies.

Collectively, our observations provide mechanistic insight into the circadian network, with *YWHAH* emerging as one of the potential endogenous circadian organizers. Furthermore, our observations suggests that *Ywhah* regulates *Cebpa* expression in subcutaneous adipose tissue (iWAT) but not significantly in visceral adipose tissue (eWAT). Thus, *YWHAH* could be a potential therapeutic target to modulate body fat distribution, a strong predictor of cardiometabolic disease [33,34]. Interestingly, even after adjusting for sex, age and BMI, liver *YWHAH* expression displayed a significant correlation with waist-to-hip ratio, an indicator of body fat distribution.

Importantly, despite the significant associations of *YWHAH* with clinical parameters and its role in modulating WAT function, the effect sizes were modest. Instead, a GRN with core clock genes as key drivers, displayed strong enrichment of genes associated with cardiometabolic health. Altogether, these observations highlight the importance of the core clock machinery in regulating physiology through pleiotropic signalling mechanisms, that are potentially controlling function in the target tissues through redundant and/or complementary mechanisms. The independent role of the individual factors controlled by the hepatocyte clock remains to be elucidated. Previously reported liver-to-WAT signals, such as FGF21, RBP4, ANGPTL4, LBP and ITIH3 [14,15], were not identified in our analyses as circadian hepatokine signals to WAT. This does not necessarily imply that these factors do not play a role in regulating circadian homeostasis. Instead, the circadian network may serve as a fine-tuning mechanism, influencing how other signals are relayed [7]. In addition, hepatokine regulation could be context-dependent and regulated by specific clock components.

For instance, the downregulation of REV-ERBα, resulting in catabolism through hepatokine signalling, has been reported in cancer cachexia [15]. Furthermore, the route of communication likely extends beyond endocrine signalling and includes neuronal communication [7,24,35]. In fact, vagus neuronal efferent projections control adipose tissue physiology through the hepatic branch [36]. Although our *in vitro* system revealed that the clock in hepatocyte temporally modulates adipocyte transcription through direct signalling, the involvement of the peripheral nervous system cannot be overruled in an *in vivo* setting. Such mechanistic insight would enable the understanding of the sufficiency, dependency, and redundancy within circadian networks [37,38]. In this study, we contribute to this knowledge by delineating the signalling from the hepatocyte clock to WAT and providing insight on the structure of the circadian network and its clinical relevance. Our findings suggest that the hepatic clock modulate WAT oscillations in coordination with the local adipose clock and/or other tissue clocks by integrating feeding-fasting cues.

Dissecting the directionality in the circadian inter-organ network will advance our understanding of how circadian rhythms are controlled and advise novel therapeutic approaches targeting the detrimental effects of circadian misalignment on metabolic homeostasis [39].

### 2.8 Limitations of the study

Emerging evidence points to sex-specific differences in circadian rhythms [40]. In this study, only male mice were included and therefore these findings cannot be extrapolated to females. This is particularly relevant in studies focusing on WAT as the depot-specific expansion of it is different between sexes [41]. Our observations of the depot-specific effects by *Ywhah* on WAT *Cebpa* expression requires further validation in females. Furthermore, the role of the hepatocyte clock in regulating WAT function through indirect mechanisms involving other organs were not investigated in this study.

Our *in vitro* system allows for investigation of direct signals from hepatocytes to differentiating adipocytes yet, the physiological relevance of these signals requires systems biology approaches considering simultaneous contributions by other endocrine organs as well as neuronal signals [7]. Future studies, involving multi-organ analyses, are required to address the complexity of the circadian network.

Our *in vitro* system provides an innovative approach to study circadian inter-organ signalling. The system facilitates genetic manipulations in either the source cells and/or the target cells in the signalling. However, our CRISPR-Cas9 mediated genetic engineering could induce off target effects, and it resulted in a partial knock out of the cell population which may introduce compensatory mechanisms by the non-engineered cell population. Furthermore, we used only 2-D cultures which introduce limitations regarding physiological relevance. This system could however be improved by implementing 3-D organoid cultures to better represent the *in vivo* situation.

The main limitation of this study is that we were unable to detect YWHAH in serum samples collected from mice. Although we confirmed its presence in extracellular vesicles (EVs) isolated from livers *ex vivo*, circulating levels may be below the detection threshold, even after EV enrichment. This raises the question of whether YWHAH acts as a direct liver-to-WAT signal or whether its effects are mediated indirectly through local mechanisms within the liver. These possibilities warrant further investigation.

Additionally, YWHAH is a ubiquitously expressed protein, and other tissues are likely involved in regulating its systemic levels. Despite this, we relied on QENIE analysis to identify liver-derived circadian factors potentially regulating WAT adipogenic gene expression. As a proof of principle, we focused on one of the predicted circadian liver genes, *Ywhah*, and validated its role *in vitro*. However, our data suggest that *Ywhah* does not regulate *Cebpa* expression in epididymal WAT *in vivo*. Nonetheless, the *in vivo* relevance of *Ywhah* remains supported by its effect on *Cebpa* expression in inguinal WAT and its clinical correlations with WAT lipid pathway genes and cardiometabolic risk factors.

Taken together, despite these limitations, our study provides robust evidence that the hepatocyte clock contributes to the regulation of transcriptional rhythms in WAT lipid metabolism.

## 3 Materials & methods

### 3.1 Animals

Mice were bred and housed in the animal facilities at the University of California–Irvine vivarium, Barcelona Science Park, Spain and, Karolinska Institutet, Sweden, in accordance with the guidelines of the Institutional Animal Care and Use Committee (IACUC) at UC-Irvine, European Union and Spanish regulations and, Stockholm South Ethical review board (Dnr:02936–2023) and the Karolinska Institute guidelines, respectively. Animal experiments were designed and conducted with consideration of the ARRIVE guidelines (Animal Research: Reporting of In Vivo Experiments). Bmal1stopFL mice were generated on the C57BL/6 J background as previously described [13,18]. Hepatocyte reconstituted (Liver-RE; hepatocyte-specific reconstitution of *Bmal1*) mice were generated by crossing *Bmal1*-stopFL with Alfp-Cre mice as previously described [18]. Experimental genotypes were - wild-type (WT) – 1. Bmal1^wt/wt^, Bmal1 knockout (KO) – Bmal1^stopFL/stop−FL^; *Bmal1* hepatocyte-reconstituted (Liver-RE) – Bmal1^stopFL/stopFL^. All experiments used 8–12-week-old male mice on a 12 h light/12-h dark cycle and all mice received diet during the dark (active) period – ZT12- ZT0.

Hepatocyte-specific *Bmal1* knockout mice (LKO) and their corresponding wild-type littermates were generated on the C57BL/6 J background. Briefly, homozygous Bmal1^lox +/+^ mice (The Jackson Laboratory, USA) were crossed with heterozygous AlfpCre^+/−^ mice to generate the experimental genotypes Bmal1-lox^+/+^, Cre^−/−^ (WT) and Bmal1-lox^+/+^, Cre^+/−^ (LKO). All experiments used 8–12-week-old male mice on a 12-h light/12-h dark cycle and received *ad libitum* diet.

Epididymal white adipose tissue (eWAT) samples from all the mouse genotypes were collected every 4 h over the circadian period. Tissues were snap-frozen in liquid nitrogen immediately after dissection and stored at −80 °C until use.

### 3.2 Metabolic phenotype of hepatocyte-specific *Bmal1*-KO mice

Hepatocyte-specific *Bmal1* knockout (LKO) and wild-type (WT) male mice, aged 8–11 weeks, fed on chow diet, and weighing between 26.06 and 29.36 g, were single housed in an indirect calorimetry system (Phenomaster, TSE systems) for a total of 10 days (240 h). Environmental conditions were maintained at 50% humidity, 22 °C and a 12:12 light–dark cycle, with lights on at 6:00 and lights off at 18:00. To minimize positional bias and ensure that cage location did not influence metabolic measurements, mice were alternately placed in metabolic cages according to genotype. The mice were divided into 2 groups with 4 biological replicates in each group (LKO; n = 4 and WT; n = 4). The data was recorded every 10 min automatically and from the total 10 days, the initial 2 days were excluded from the analysis since it was considered as an acclimatisation period. Data analyses were performed using Microsoft Excel and Prism 10.1 (GraphPad). The average hourly value was calculated for each 10-minute recording of locomotor activity, energy expenditure, respiratory exchange ratio, and food intake.

### 3.3 Oral glucose tolerance test

Hepatocyte-specific *Bmal1* knockout (LKO) and wild-type (WT) male mice on chow diet aged 8–11 weeks were used (LKO; n = 4, WT; n = 4). Mice were fasted for 6 h before a glucose bolus (1 g/kg body weight) was administered orally via gavage. The body weight was recorded before the administration to calculate the appropriate dose. Blood glucose levels were measured at 0 (prior to glucose administration), 15, 30, 60 and 120 min from a tail cut after glucose administration using a glucometer (AccuCheck instant, Roche).

### 3.4 Cell culture and differentiation

AML12 and 3T3-L1 cells were cultured according to previously published protocols [25,42]. AML12 cells were cultured in 10% fetal bovine serum in growth media comprised of Dulbecco's Modified Eagle Medium/Nutrient Mixture F-12, HEPES, 100 U/mL Penicillin/streptomycin, 1% Insulin-Transferrin-Selenium solution (Gibco) and 40 ng/mL dexamethasone. Cells were grown until 90% confluence was reached. To synchronise the circadian clock, we washed the cells with PBS and replaced it with media containing 100 nM dexamethasone for 1-h followed by another PBS wash and incubation with dexamethasone-free media 3T3-L1 cells were cultured in 10% bovine calf serum in growth media consisting of DMEM, 2 mM l-glutamine, 100 U/ml penicillin, and 100 U/ml streptomycin. Once cells reached confluence, we used a standard DMI protocol to induce differentiation: cells were treated with a medium containing a commonly used DMI (dexamethasone/IBMX/insulin) stimulus and rosiglitazone (BRL) to initiate adipogenesis. Applying the DMI stimulus consisted of replacing the media on the cells with growth media plus 10% FBS, 100 μM IBMX (Sigma Cat # 7018), 1 μM dexamethasone (Sigma Cat #D1756), 1.75 nM bovine insulin (Sigma Cat #I6634) and 10 uM rosiglitazone (Cayman Chemicals Comp. USA, #71740). Three days after initiating differentiation, the media was removed and was replaced with growth media plus 10% FBS and 1.75 nM insulin for two more days.

### 3.5 3T3-L1 synchronization

3T3-L1 WT and *Bmal1*-KO cells were synchronized following the protocol described by Pendergrast et al. [43]. Briefly, cells were seeded in 24-well plates and allowed to proliferate for 48 h until reaching approximately 90% confluency. Differentiation was then initiated using standard medium supplemented with DMI and BRL for three days. On day 4, the medium was replaced with standard medium containing bovine insulin for 24 h. Afterwards, cells were washed once with PBS and synchronized with 200 nM dexamethasone for 30 min, followed by a single wash with differentiation medium. The end of the dexamethasone treatment was designated as Cell-Time (CT) 0. Cells were then maintained in differentiation medium containing bovine insulin. Starting 12 h post-synchronization, cells were harvested every 4 h for RNA processing.

### 3.6 Human data

The STARNET study is described in detail in the 2016 Science [30] and 2022 Nature Cardiovascular Research [31] publications. To date, blood, metabolic and arterial wall biopsies have been isolated from a total of 1,447 patients with obstructive CAD during coronary artery bypass surgery (CABG) and uniquely, 434 non-CAD controls without obstructive CAD (as verified in preoperative angiogram with SYNTAX score = 0) during other forms of open-thorax surgeries such as valve replacements (Figure 1). All patients gave written informed consent (ethical approval: Dnr: 385/M−1 (2023)) Patients with other active severe systemic disease (e.g., inflammatory disease or cancer) were excluded. Samples were collected in the mornings after an overnight fast. The primary endpoints are the extent of coronary atherosclerosis (SYNTAX score in preoperative angiograms. Each participant completed a questionnaire to assess disease history, current drug regimen, and lifestyle (e.g., daily activity, alcohol consumption, smoking) and was screened for 114 clinical variables including lab. chemistry. >95% are whites, 31% females; 32% have diabetes, 75% have hypertension, 67% have hyperlipidemia; and among the cases, 33% had an MI before age 60. Bulk RNAseq data from 800 CAD cases and 250 nonCAD controls have been generated using the HighSeq2000 platform to a depth of 25–40 million reads, mostly with the Ribo-Zero protocol. DNA samples from these cases and controls were genotyped (n = 934/215) with either the OmniExpress Exome array (Illumina, ∼900 k SNPs) or the Illumina's Infinium Global Screening Array 24v1 platform and imputed to a total of 11 million variant calls. In 2020, blood DNA samples were whole-genome sequenced (BGI, DNBSEQ500, 40 M read depth, 150/100 PE, n = 942/204). Herein, regression analyses, adjusting for age, sex and BMI, were performed on liver *YWHAH* and liver core clock genes or the visceral white adipose tissue transcriptome. Gene regulatory networks (GRNs) controlled by the liver clock were identified by assessing the enrichment of clock-controlled transcripts (genes rhythmic in the livers from WT and LRE but not KO mice) in the STARNET cohort.

### 3.7 RNA extraction

RNA from cells was extracted using commercial kits (MACHEREY-NAGEL GmbH & Co. KG) according to the manufacturer's instructions. For tissues, they were soaked in Qiazol Lysing Reagent (Qiagen, Germany) and homogenized in a Bead Ruptor with stainless steel beads and RNA was isolated using RNeasy lipid tissue mini kit (Qiagen, Germany) according to manufacturer's instructions. The concentration, quality and purity were measured using Nanodrop 2000 (Thermo Fisher Scientific, Lafayette, CO).

### 3.8 qRT-PCR

500 ng of tissue RNA and 200 ng of RNA isolated from cells were reverse transcribed with iScript cDNA synthesis kits (Bio-Rad, Hercules, CA). Quantitative real-time polymerase chain reaction (qRT-PCR) analysis was performed using CFX Opus 96.

Real-Time PCR System (Bio-Rad, Hercules, CA) with SYBR Green Master Mix.

(Applied Biosystems). *Cebpα* (Fwd – CAAGAACAGCAACGAGTACCG, Rev -

GTCACTGGTCAACTCCAGCAC), *Arnlt* (Fwd – GCAGTGCCACTGACTACCAAGA, Rev - TCCTGGACATTGCATTGCAT), *Per2* (Fwd -CGCCTAGAATCCCTCCTGAGA,

Rev - CCACCGGCCTGTAGGATCT), *Dbp* (Fwd – GGAAACAGCAAGCCCAAAGAA,

Rev - CAGCGGCGCAAAAAGACTC), *Nr1d1* (Fwd -TACATTGGCTCTAGTGGCTCC,

Rev - CAGTAGGTGATGGTGGGAAGTA), *Ywhah* (Fwd –

ACGAAGATCGAAATCTCCTCTCT, Rev - CCGGTAGGCTTTAACTTTCTCCA) and normalized against the housekeeping genes *18s* (Fwd –

CGCCGCTAGAGGTGAAATTC, Rev - CGAACCTCCGACTTTCGTTCT), *Lrp10* (Fwd.

– GGATCACTTTCCCACGTTCTG, Rev - GAGTGCAGGATTAAATGCTCTGA) and.

*Hprt* (Fwd – TCAGTCAACGGGGGACATAAA, Rev -

GGGGCTGTACTGCTTAACCAG).

### 3.9 RNA sequencing for WAT samples and AML12 cells

Mouse WAT samples and cultured AML12 hepatocytes were sequenced using the Smart-seq3 method as previously described [44]. Briefly, RNA was denatured at 72 °C for 10 min. To each sample, 1 μL of a reverse transcription mix (25 mM Tris–HCL, pH 8.3; 30 mM NaCl; 1 mM GTP; 2.5 mM MgCl2; 8 mM DTT; 0.5 U μl recombinant RNase inhibitor, 2 μM template-switching oligo (5′-biotinAGAGACAGATTGCGCAATGNNNNNNNNrGrGrG-3′)) and 2 U μl−1 of Maxima H- minus reverse transcriptase enzyme was added. Afterwards, samples were reverse transcribed and the reaction was terminated by incubating at 85 °C for 5 min. Directly after, PCR pre-amplification was performed by adding 6 μL of PCR mix, bringing reaction concentrations to 1 × KAPA HiFi PCR buffer (Roche), 0.3 mM dNTPs, 0.1 μM Smartseq3 forward PCR primer (5′- TCGTCGGCAGCGTCAGATGTGTATAAGAGACAGATTGCGCAATG-3′; IDT) and 0.1 μM Smartseq3 reverse PCR primer (5′-ACGAGCATCAGCAGCATACGA-3′; IDT)). All samples were purified with AMpure XP beads (Beckman Coulter). To check for library size distribution, a high-sensitivity DNA chip (Agilent Bioanalyzer) was used. cDNA concentrations were quantified using the Quant-iT PicoGreen dsDNA Assay Kit (Thermo Scientific). Subsequently, the libraries were pooled sequenced at the 50-bp single end using an Illumina NextSeq500 instrument.

Raw non-demultiplexed fastq files were processed using zUMIs (version 2.4.1 or newer) with STAR (v2.5.4 b) to generate expression profiles for both the 5′ ends containing UMIs as well as combined full-length and UMI data. To extract and identify the UMI-containing reads in zUMIs, find_pattern: ATTGCGCAATG was specified for file1 as well as base_definition: cDNA (23–75; single end), (23–150-bp, paired end) and UMI (12–19) in the YAML file. Mouse cDNA was mapped against mm10 genome with CAST SNPs masked with N to avoid mapping bias. Data were quantified with gene annotations from Ensembl GRCm38.91.

### 3.10 CRISPR-mediated gene silencing

Single-guide RNAs (sgRNAs) targeting *Arntl* and *Ywhah* were designed using the CRIPR design tool (publicly available at https://chopchop.cbu.uib.no/). We chose the guides offering both the highest rank in terms of efficiency and lowest off – target effects for both *Arntl* (sgRNA - GCATCAATGAGTCGCTCCCGGGG) and *Ywhah* (sgRNA -

TGAAGGCGGTGAGCGCGCTGGGG). A total of 6uM of recombinant *Streptococcus pyogenes* Cas9 (Integrated DNA Technologies, Inc.) protein was pre – incubated with 12uM of respective sgRNA 30 min at room temperature before transfection.

Proliferating AML12 and 3T3-L1 cells were then transfected using Neon Transfection.

System 100 μL Kit (Invitrogen) with the following conditions: 1700 V, 20 ms, 1 pulse. Validation of gene knockdown was assessed by qPCR and Western blot (as described below).

### 3.11 AML12 conditioned media stimulation of 3T3-L1 cells

To study cross-cell communication *in vitro*, AML12 WT, *AML12 Bmal1*-KO and AML12 *Ywhah*-KO cells were cultured at a density of 5 × 10^5^ cells per well in a 24-well plate until 90 % confluence was reached. Afterwards, cells were exposed to media containing 100 nM dexamethasone for 1 h to synchronise the clocks followed by dexamethasone starvation throughout the sample collection. After 12 h, 3T3-L1 differentiation medium containing 10% FBS and 1.75 nM bovine insulin was put on the synchronised AML12 cells for 4 h to capture the released hepatokines within the time windows CT12-16, CT16-20, CT20-24, CT24-28, CT28-32 and, CT32-36.

To study the effect of the secreted hepatic factors on pre-adipocytes, 3T3-L1 cells were first cultured in 48-well plates and cells were differentiated for 3 days with standard medium containing DMI and BRL, as described in the previous section. Thereafter, cells were washed with PBS, then cultured for 6 h with the conditioned media collected from the AML12 cells. For experiments investigating the effects of hepatokines, media were treated with Proteinase K (20 mg/mL), then boiled at 50 °C for 45 min. To inactivate proteinase K, media was subsequently heated with 1 mM Ethylenediaminetetraacetic acid (EDTA) for 15 min at 80 °C. Control samples were also exposed to EDTA and heated to 80 °C.

Total protein content was determined using silver staining (Pierce silver stain kit, Thermo Fisher, Cat. No. 24612) of the SDS PAGE gel as well as Ponceau S staining of the PVDF membrane after transfer.

### 3.12 Overexpression experiments

Messenger RNA (mRNA) for *Ywhah* was synthesized using the HiScribe T7 ARCA mRNA Kit according to the manufacturer's protocol (New England Biolabs Inc.). Briefly, template DNA for the *in vitro* transcription reaction (IVT) was prepared by PCR using complementary DNA (cDNA) from AML12 cells as a template. The PCR primers contained a T7 promoter sequence and are as follows: Forward (5′ACACTAATACGACTCACTATAGGGGCCACCATGGGGGATCGAGAGCAGCT-3′, Reverse (5′-TCAGTTGCCTTCTCCTGCTTCTTC-3′). The PCR mixture consisted of 0.5 μM primers, 10 mM dNTPs, Q5 Reaction Buffer (New England Biolabs Inc.), Q5 Hot Start High-Fidelity DNA Polymerase (New England Biolabs Inc.) and nuclease free water. The mixture was subjected to the thermal cycling reaction as follows: 98 °C, 30 s → (98 °C, 10 s → 69 °C, 30 s →72 °C, 70 s) × 35 cycles → 72 °C, 2 min. The amplification was analyzed by agarose gel electrophoresis, and the PCR product was purified using the PCR clean-up Gel extraction kit (MACHEREY-NAGEL GmbH & Co. KG). 1 μg of DNA (PCR product) was used in a IVT reaction containing 5 mM DTT, 2x ARCA/NTP mix, T7 RNA Polymerase mix and nuclease free water. After the mixture was incubated at 37 °C for 30 min, DNAse I (New England Biolabs Inc.) was added to the mixture at a final concentration of 0.1 units/μL and was further incubated at 37 °C for 15 min. Subsequently, a Poly(A) tailing reaction was set up containing the unpurified IVT reaction, Poly(A) Polymerase Reaction buffer and Poly(A) Polymerase (New England Biolabs Inc.) and further incubated at 37 °C for 30 min. Synthesized mRNA was purified using the Monarch Spin RNA Cleanup Kit (New England Biolabs Inc.) according to the manufacturer's instructions. RNA concentration was measured by a Nanodrop 2000 (Thermo Fisher Scientific, Lafayette, CO). Differentiating 3T3-L1 cells were then transfected using Neon Transfection System 100 μL Kit (Invitrogen) with the following conditions: 1700 V, 20 ms, 1 pulse. Validation of Ywhah overexpression was assessed by qPCR.

*In vivo Ywhah* overexpression was performed by encapsulating the mRNA in lipid nano particles (LNP) from *in vivo*-jetRNA®+ (Sartorius, USA) according to manufacturer's instructions. The mRNA-LNP mixture was injected intravenously through the tail in 9week-old C57BL/6 J male mice at ZT8. Each mouse received 0.5 μL/g body weight from the mRNA-LNP mixture consisting of 0.1 μg mRNA/μL. After 24 h, mice were sacrificed, and tissues were collected and snap-frozen in liquid nitrogen immediately after dissection and stored at −80 °C until use.

### 3.13 Western blot analyses

Western blots were performed as previously described [45]. Briefly, RIPA buffer (50 mM Tris–HCl [pH 8.0], 150 mM NaCl, 1% NP-40, 0.5% Sodium Deoxycholate, 0.1% SDS, 5 mM MgCl2, and 1 mM PMSF) supplemented with Protease Inhibitor Cocktail (Roche) supplemented with and phosphatase inhibitors (Millipore Sigma) was used to lyse frozen tissue samples and cells, which were then heated at 95 °C for 5 min with a denaturation buffer. Proteins were separated by SDS–PAGE electrophoresis and transferred to PVDF membranes (Amersham International, GE Healthcare). Membranes were incubated in blocking reagent (3% of Amersham ECL Prime Blocking solution reagent in Tris-buffered saline-tween 20 (TBS-tween)) for 1 h, then in primary antibody overnight at 4 °C. The antibodies used were anti-BMAL1, Abcam, ab93806; anti-YWHAH, Cell Signalling, #9640;- anti-SYNTENIN, Abcam, ab19903; anti-alphaTUBULIN, Cell Signalling, #9099; anti-β-ACTIN, Sigma Aldrich, a5441; anti-H3, Cell.

Signalling, #14269; Albumin, Bethyl Laboratories, #A90-134 A; anti-TSG101, Abcam #EPR7130(B); anti-CD63, Abcam, #EPR5702, anti-CD81, Abcam, ab109201; antiP84, Genetex, GTX70220 and LAMIN A/C, Cell Signalling, #4777. After three washes in TBS-tween (10 min each), membranes were incubated in Anti-mouse IgG, HRP conjugate, EMD Millipore, AP160P or Anti rabbit IgG, HRP linked, Cell Signaling Technologies, 7074 S for 1 h at room temperature in 3% blocking solution. Membranes were incubated with ECL western-blotting substrate (Amersham International, GE Healthcare) and images were acquired using a Chemidoc XRS system or ChemiDOC (Bio-Rad Laboratories).

### 3.14 Western blot analyses for conditioned medium-derived EVs

EVs were lysed using lysis buffer, and 50 μg of total protein was loaded onto a NuPAGE™ 4–12% Bis-Tris protein gel (1.5 mm, NP0335). Electrophoresis was performed at 200 V for 30 min, followed by transfer onto a nitrocellulose membrane using iBlot™ 2 Transfer Stacks. The membrane was blocked with Intercept™ (TBS) Blocking Buffer for 1 h at room temperature, then incubated overnight at 4 °C with the following primary antibodies: anti-Alix (EnkiLife, APRab00291, 1:500), anti-CD63 (EnkiLife, AMRe01577, 1:500), and anti-TSG101 (EnkiLife, AMRe02725, 1:500). After washing with TBST, the membrane was incubated with Goat Anti-Rabbit IgG (IRDye® 680RD, 1:5000) for 2 h at room temperature. Imaging was performed using a BioRad gel documentation system.

### 3.15 Isolation of liver-derived small extracellular vesicles

Eight-week-old C57/BL6J mice were anesthetized by isoflurane, the hepatic portal vein was clipped with scissors, and the inferior vena cava was cannulated using a butterfly needle for perfusion at 2 mL/min with PBS. Following perfusion, liver lobes were removed, placed in a 50 mL conical tube, and bubbled with carbogen in chemically defined, CHO media. Following a 45-minute incubation period, the supernatant was collected and centrifuged at 2,000 g for 5 min at 4 °C to pellet cellular debris. This supernatant was then mixed with Thermo Exosome Isolation Reagent (Invitrogen Ref #4478359) following the manufacturer's protocol to precipitate extracellular vesicles. Briefly, following an overnight incubation at 4 °C, the mixture was centrifuged at 10,000 g for 1 h at 4 °C, followed by resuspension in PBS and re-centrifugation at 10,000 g for 1 h at 4 °C. Extracellular vesicles were then resuspended in an appropriate volume of RIPA buffer including protease and phosphatase inhibitor cocktail for downstream applications. Total protein content was determined using zinc staining (Pierce Zinc Reversible Stain Kit, Thermo Fisher, Cat. No. 24582) of the SDS PAGE gel.

### 3.16 Transmission Electron Microscopy (TEM)

Three microliters of the sample were applied to glow-discharged, carbon-coated, formvar-stabilized 400 mesh copper grids (easiGlow™, Ted Pella) and incubated for approximately 30 s. The excess sample was then blotted off, followed by a MilliQ water wash. Grids were negatively stained with 1% ammonium molybdate and imaged using an HT7800-Xarosa transmission electron microscope (Hitachi HighTechnologies) operated at 80 kV, equipped with a 4 MP Veleta CCD camera (Olympus Soft Imaging Solutions GmbH).

### 3.17 Nanoparticle Tracking Analysis (NTA)

Nanoparticle tracking analysis was performed using the ZetaView PMX-220-12H-R5 instrument (Particle Metrix) in both scatter and fluorescence mode (488 nm). Calibration beads and biological samples were diluted in PBS to a final volume of 1 mL. For calibration, 100 nm polystyrene beads were diluted 1:250,000 (v/v). EV samples were captured with the following settings: sensitivity 80, shutter speed 100, and cell temperature maintained at 25 °C. Measurements were preceded by a PBS wash to prevent sample carryover. For EVs isolated by size exclusion chromatography (SEC), two imaging cycles were performed by scanning 11 cell positions. Video capture was conducted at 30 frames per second. Data analysis was done using ZetaView software version 8.06.01, with the following parameters: minimum brightness 25, minimum area 10, and maximum area 200.

### 3.18 Size exclusion chromatography (SEC)

AML12 conditioned media was first filtered through a 0.22 μm filter and then concentrated using a 10 KDa spin filter. The concentrated media was processed through 70 nm Izon qEV original Gen2® columns. After discarding the initial 3 mL void volume, EV fractions 4–5 were collected separately and concentrated with a 10 KDa spin filter. Fractions 7–12 were pooled, with 2 mL collected for each, to collect protein molecules. All fractions were analyzed using the Zeta View Nanoparticle Tracking Analyzer (Particle Metrix GmbH) and Pierce® BCA protein assay kit (Thermo Scientific, Cat. No 23235) to quantify EVs and proteins. Absorbance was measured by CLARIOstar® Plus (BMG LabTech).

### 3.19 Cryogenic electron microscopy (cryo-EM)

EV's were sonicated at the ultra-low setting of the Diagenode Bioruptor three times for 10 s each in PBS for CryoEM preparations. 3 μL of the EV preparation was applied to lacey carbon grids (4–6 nm thick on 300 mesh Gold grid, Catalog No. 50193–1309, Electron Microscopy Sciences) that had been glow discharged at 20 mA for 30 s in a Quorum EMS glow discharger. Grids were vitrified with a Vitrobot Mark IV (Thermo Fisher Scientific) maintained at 8 °C and 100% humidity. A blot force of 0 and blot time of 3 s was applied before plunge freezing into liquid ethane. The data were collected at 200 kV on a Glacios cryo-transmission electron microscope equipped with a Falcon IV camera and Selectris energy filter (slit width: 10 eV) at the University of Texas Health San Antonio Cryo-EM Facility. Imaging was acquired using Thermo Fisher Scientific's EPU software. The total dose was 53 e−/Ǻ2, with a −2 nm defocus. Micrographs were recorded at either 130,000X or 63,000x with a pixel size of 0.19 nm and 0.15 nm, respectively. In all, 100 non-overlapping micrographs were taken to confirm size and morphology of sEV preparation.

### 3.20 Dynamic Light Scattering (DLS)

To measure the diameter of sEV's, the Diagenode Bioruptor was used to gently sonicate for 10 s on the ultra-low setting three times to break up aggregates. The DynaPro NanoStar machine was turned on for at least 30 min prior to the beginning of the experiment. Cuvettes were cleaned using soapy water, followed by ethanol, and were thoroughly dried with compressed air to eliminate presence of dust particles. Extracellular vesicles were diluted by a factor of 1:10 in filtered (0.22 μm) PBS to allow for proper dispersion of particles. The laser wavelength was set to 663.87 μm, temperature was controlled to room temperature and the peak radius cutoff was set between 0.5 nm and 5,000 nm. The auto attenuation limit was set to 60s and the wait time was 5 min. Laser intensities were set to 25%. Prior to running sEV particles, 50 μL of a known standard, ubiquitin, was run as an internal control and peaks were observed at 1.6–1.8 nm. 50 μL of dilute sEV sample was run to measure diameter of EV particles.

### 3.21 Seahorse assay

Oxygen consumption rates (OCR) were measured with XF96 Seahorse Extracellular Flux Analyzer (Agilent) using Cell Mito Stress Test kits (Agilent Technologies, USA). To assess basal OCR, cells were pre - incubated in Sea Horse medium (Agilent Technologies, USA) supplemented with 1 mmol l^−1^ pyruvate, 2 mmol l^−1^ glutamine and 10 mmol l^−1^ glucose. Then, Mito Stress assay was performed by sequential addition of 1.5 μmol l^−1^ oligomycin (inhibitor of ATP synthesis), 1.5 μmol l^−1^ FCCP (uncoupling agent) and 0.5 μmol l^−1^ rotenone/antimycin A (inhibitors of complex I and complex III of the respiratory chain, respectively). For estimation of the basal and maximal respiration, the mean non-mitochondrial respiration was subtracted from the mean values of basal and maximal respiration.

### 3.22 Assessment of lipid area

3T3-L1 pre-adipocytes were plated in Corning® CellBIND® 96 well plates up to 90% confluence. Thereafter, medium was switched to AML12 conditioned media for the following 7 days of differentiation, with daily changes. Cells were then fixed on the plates using 4% paraformaldehyde for 15 min and stained with Hoechst and BODIPY for 20 min, then preserved in PBS. High – throughput quantification of nuclear (Hoechst) and lipids (BODIPY) staining were carried out using a CellInsightCX5 High Content Screening Platform at 10X magnification.

### 3.23 Bioinformatic analyses

To identify oscillating transcripts, transcriptomic data for liver, WAT and AML12 cells were analyzed using the DryR package [19].

For the WAT and liver tissues, raw count data were input into the *dryseq* function using the default settings which performs median-of-ratios normalization as implemented in DESeq2. Lowly expressed genes (sum of normalized counts <10 across samples) were filtered out. Principal component analysis (PCA) was used to assess the presence of batch effects; samples clustered primarily by treatment and timepoint, with no evidence of batch-driven separation. As such, batch correction was not applied in the model. Rhythmicity was assessed, with a 24-hour period specified, by fitting rhythmic, flat, and other time-dependent models. Model selection was based on Bayesian Information Criterion weights (BICW), which represent the posterior probability of each model. To ensure robust model assignment, we repeated the analysis after excluding genes with a maximum BIC weight below 0.4; this filtering did not alter the overall conclusions, and therefore all genes assigned to each model were included in the final analysis.

For the AML12 RNA-seq data, raw counts were normalised by dividing each gene count by the mean count across all genes within each sample. While this approach differs from the DESeq2 normalization used for tissue data, it was selected to better preserve subtle temporal expression changes characteristic of *in vitro* systems. Rhythmicity of the normalized data was analysed as described above, except that the drylm function was used instead, facilitating rhythmic analyses using linear models.

For *in vivo* and *in vitro* experiments that were not generated using omics, rhythmicity with a circadian period of 20–28 h were detected using CosinorOnline (https://cosinor.online/app/cosinor.php). Genes were considered significantly circadian if their permutation-based, adjusted P value was <0.05.

For pathway analyses we used the online ToppFun tool (https://toppgene.cchmc.org/enrichment.jsp) for gene enrichment analyses. Biological pathways with a p-value <0.05 and were selected.

### 3.24 Quantitative endocrine network interaction estimation (QENIE)

To quantify and predict the impact of Liver genes on global gene expression in WAT, we applied a bioinformatics framework named QENIE using data from the hybrid mouse diversity panel [27,46,47]. Briefly, this approach was used to screen for strong Liver-WAT correlations by aggregating significance levels of global transcript correlations across organs (Ssec) for each gene in the origin tissue (Liver) with rhythmic DEG (WAT) transcripts. The underlying data used is derived from ∼100 genetically diverse strains of mice which were all sacrificed between 9 and 11AM42,43. To generate correlation co-efficient and associated P statistic, the R package WGCNA *bicorAndvalue*(.) function was applied.

### 3.25 Statistics

Data were analysed using Prism 10.1 (GraphPad) and RStudio. Graphs display mean values, and error bars represent the standard error of the mean. Significant circadian transcripts across all RNA-sequencing datasets were identified using the dryR method in RStudio (see Methods for details). The dryR output, including detailed statistics for each transcript's circadian rhythm, is provided in the supplementary table corresponding to each figure. For panels assessing rhythmicity of non-omics data, cosinor p-values are reported. The number of biological replicates for each experiment is indicated in the figure legends. Statistical comparisons between groups at individual time points were performed using unpaired Student's t-tests, ordinary one-way ANOVA, or two-way ANOVA followed by Fisher's LSD test. All significance values are reported in the figure legends.

## CRediT authorship contribution statement

**Ivan Vlassakev:** Writing – review & editing, Visualization, Validation, Investigation, Formal analysis, Data curation. **Christina Savva:** Writing – review & editing, Visualization, Investigation, Data curation. **Gianluca Renzi:** Writing – review & editing, Investigation, Data curation. **Hema S. Ilamathi:** Writing – review & editing, Investigation, Formal analysis, Data curation. **Doste R. Mamand:** Writing – review & editing, Formal analysis, Data curation. **Jacob G. Smith:** Writing – review & editing, Investigation, Funding acquisition. **Carolina M. Greco:** Methodology, Investigation. **Christopher Litwin:** Visualization, Investigation, Data curation. **Qing Zhang:** Investigation, Data curation. **Leandro Velez:** Writing – review & editing, Software, Data curation. **Angela Ma:** Visualization, Data curation. **Martin O. Bergo:** Writing – review & editing, Resources, Methodology. **Oscar P.B. Wiklander:** Writing – review & editing, Supervision, Resources. **Pura Muñoz-Cánovez:** Writing – review & editing, Supervision. **Niklas Mejhert:** Writing – review & editing, Supervision. **Marcus Seldin:** Writing – review & editing, Software, Resources, Methodology, Data curation. **Johan L.M. Björkegren:** Writing – review & editing, Formal analysis, Data curation. **Paolo Sassone-Corsi:** Conceptualization. **Kevin B. Koronowski:** Writing – review & editing, Validation, Supervision, Methodology, Funding acquisition, Formal analysis, Data curation. **Salvador Aznar Benitah:** Writing – review & editing, Supervision, Resources, Investigation, Funding acquisition. **Paul Petrus:** Writing – review & editing, Writing – original draft, Supervision, Project administration, Methodology, Investigation, Funding acquisition, Formal analysis, Data curation, Conceptualization.

## Declaration of competing interest

The authors declare the following financial interests/personal relationships which may be considered as potential competing interests:Johan Bjorkegren reports a relationship with Clinical Gene Networks AB (CGN) that includes: equity or stocks. If there are other authors, they declare that they have no known competing financial interests or personal relationships that could have appeared to influence the work reported in this paper.

## Data Availability

Data will be made available on request.

## Appendix A Supplementary data

Multimedia component 5 Multimedia component 5

Figure S1 a) Validation of BMAL1 knockdown in liver tissue by qPCR and Western blot. WT (n = 24), LKO (n = 24), four biological replicates per time point. Western blot: WT (n = 3), LKO (n = 4). The graph represents mean values of the relative gene expression and error bars represent standard error of the mean. Cosinor *p*-values are shown; statistical comparisons between groups at individual time points were performed using two-way ANOVA with Fisher's LSD test. ∗p-value <0.05 ∗∗ p-value <0.01, ∗∗∗∗p-value <0.0001. b-c) Body weight and adipose tissue mass as % of total body weight. Four biological replicates per genotype were sampled. d) 24-hour food intake (light vs dark). The graph represents mean values and error bars represent standard error of the mean, four biological replicates per group. Statistical comparisons between the light and dark period were performed using two-way ANOVA with Fisher's LSD test. ∗∗∗∗p-value <0.0001. e-f) Locomotor activity in light–dark conditions. Four biological replicates per genotype were sampled. Statistical comparisons between the light and dark period were performed using two-way ANOVA with Fisher's LSD test. ∗∗∗∗p-value <0.0001. g-h) Respiratory exchange ratio (RER) in light–dark conditions. Four biological replicates per genotype were sampled. Statistical comparisons between the light and dark period were performed using two-way ANOVA with Fisher's LSD test. ∗∗∗∗p-value <0.0001. i-j) Energy expenditure (EE) measurement in light–dark conditions. Four biological replicates per genotype were sampled. Statistical comparisons between the light and dark period were performed using two-way ANOVA with Fisher's LSD test. ∗∗∗∗p-value <0.0001. k-l) Oral glucose tolerance test curves for WT (black) and LKO (red) mice and comparison of areas under the curve (AUC) between the genotypes. Four biological replicates per genotype were sampled at each time point. Statistical comparisons between groups at individual time points for panel k were performed using two-way ANOVA with Fisher's LSD test. ∗ p-value <0.05, ∗∗ pvalue <0.01, ∗∗∗p-value. A student unpaired t-test was performed to determine significant differences between groups for panel l. ∗∗ p-value <0.01. m) qPCR relative expression of core clock genes in WAT from WT (black) and LKO (red) mice (n = 30 per genotype; five biological replicates per time point). Graphs represent mean values of the relative gene expression and error bars represent standard error of the mean. Cosinor *p*-values are shown; statistical comparisons between groups at individual time points were performed using two-way ANOVA with Fisher's LSD test. ∗∗ p-value <0.01, ∗∗∗p-value <0.001. n) Western blot image of BMAL1 and its relative protein abundance in WAT from WT and LKO mice. Outliers were identified using the Robust regression and Outlier removal (ROUT). At least two biological replicates per genotype were sampled at each time point. The graph represents mean values and error bars represent standard error of the mean. Cosinor *p*-values are shown. Figure S2. a) qPCR relative expression of core clock genes in WAT from WT (black), KO (red) and LRE (blue) mice (n = 24 per genotype; four biological replicates per time point). Graphs represent mean values of the relative gene expression and error bars represent standard error of the mean. Cosinor *p*-values are shown; statistical comparisons between groups at individual time points were performed using two-way ANOVA with Fisher's LSD test. ∗Represents significant differences in the comparison between WT and KO and # represents significance in the comparison between WT and LRE. ∗∗ p-value <0.01, ∗∗∗p-value <0.001, ∗∗∗∗p-value <0.0001, ^#^p-value <0.05, ^###^p-value <0.001, ^####^p-value <0.0001. b) Log2 expression of murine spermatogenesis genes rhythmic in WT (black), but not in KO (red) and LRE (blue) mice (n = 24 per genotype; four biological replicates per time point). Graphs represent mean log2 expression values and error bars represent standard error of the mean. Statistical comparisons between groups at individual time points were performed using two-way ANOVA with Fisher's LSD test. ∗Represents significant differences in the comparison between WT and KO and # represents significance in the comparison between WT and LRE. ∗∗∗∗p-value <0.0001, ^####^p-value <0.0001. c) UMAP of single-cell RNAsequencing from human WAT showing expression of spermatogenesis-related genes. T lymphocytes (T), natural killer cells (NK), natural killer T cells (NKT), monocytes (mono), macrophages (macro), B lymphocytes (B), mast cells (mast), fibro-adipogenic progenitors (FAPs). d) Log2 expression of *Adipor1*, *Apoe* and *Ldlr* in WAT from WT (black), KO (red) and LRE (blue) mice (n = 24 per genotype; four biological replicates per time point). Graphs represent mean log2 values and error bars represent standard error of the mean. Statistical comparisons between groups at individual time points were performed using two-way ANOVA with Fisher's LSD test. # represents significance in the comparison between WT and LRE and ‡ represents significance in the comparison between KO and LRE. ^##^p-value <0.01, ^‡^p-value <0.05. Figure S3. a) BMAL1 protein levels against A/C LAMIN in AML12 WT and *Bmal1*-KO cells. Number of biological replicates for each group is eight. A student unpaired t-test was performed to determine significant differences in protein levels. ∗∗∗∗ p-value <0.0001. b) Trypan blue viability assay of AML12 WT and *Bmal1*-KO cells. Four biological replicates per genotype were sampled. c) qPCR expression of core clock genes in synchronised AML12 WT (n = 24) and *Bmal1*-KO cells (n = 24). Four biological replicates per genotype were sampled at each time point. The graph represents mean values of the relative gene expression and error bars represent standard error of the mean. Cosinor *p*-values are shown; statistical comparisons between groups at individual time points were performed using two-way ANOVA with Fisher's LSD test. ∗p-value <0.05, ∗∗ p-value <0.01, ∗∗∗p-value <0.001, ∗∗∗∗p-value <0.0001. d) Trypan blue viability assay of 3T3-L1 WT and *Bmal1*-KO cells treated with different conditioned media for 6 h. Four biological replicates for each group were sampled. As control, cells were treated with naive medium. Statistical significance between groups was determined using a two-way ANOVA with Fisher's LSD test. ∗p-value <0.05, ∗∗ p-value <0.01. e) Schematic representation of the *in vitro* experimental approach (Created with BioRender.com). f) Western blot image of BMAL1 and the reference protein P84 in unsynchronized proliferating 3T3-L1 WT and *Bmal1*-KO cells. Six biological replicates per genotype were sampled. A student unpaired t-test was performed to determine significant differences in protein levels. ∗∗∗∗ p-value <0.0001. g) qPCR analyses of *Cebpa* in 3T3-L1 WT (n = 36) and *Bmal1*-KO cells (n = 46) treated with naive (n = 8) and conditioned medium from AML12 WT cells for 6 h. Outliers were identified using the Robust regression and Outlier removal (ROUT). Number of biological replicates for each timepoint is minimum six. The graphs represent mean values of the relative gene expression and error bars represent standard error of the mean. Cosinor *p*-values are shown. h) Relative expression of *Bmal1* in synchronised 3T3-L1 WT (n = 36) and *Bmal1*-KO cells (n = 35). Outliers were identified using the Robust regression and Outlier removal (ROUT). At least five biological replicates for each group and timepoint were sampled. The graph represents mean values of the relative gene expression and error bars represent standard error of the mean. Cosinor p-values are shown; statistical comparisons between groups at individual time points were performed using two-way ANOVA with Fisher's LSD test. ∗∗∗∗ p-value <0.0001. i) Relative expression of *Cebpa* in synchronised 3T3-L1 WT (n = 34) and *Bmal1*-KO cells (n = 36). Outliers were identified using the Robust regression and Outlier removal (ROUT). At least four biological replicates for each group and timepoint were sampled. The graph represents mean values of the relative gene expression and error bars represent standard error of the mean. Cosinor *p*-values are shown; statistical comparisons between groups at individual time points were performed using two-way ANOVA with Fisher's LSD test. ∗p-value <0.05, ∗∗ p-value <0.01, ∗∗∗p-value <0.001, ∗∗∗∗p-value <0.0001. j) Images of 3T3-L1 WT cells at 10X magnification. Hoescht (Nuclei, blue) and Bodipy (lipid droplet, green) staining of differentiated 3T3-L1 adipocytes treated with conditioned medium from AML12 WT and *Bmal1*-KO cells for 7 days, throughout the differentiation period. The scale bars on all images are 100 μm. k) Oxygen consumption rate of 3T3-L1 adipocytes treated with conditioned medium from AML12 WT (n = 48) or *Bmal1*-KO cells (n = 48) for 6 h. Number of biological replicates for each timepoint is eight. Graphs represent mean values and error bars represent standard error of the mean. Statistical comparisons between groups at individual time points were performed using two-way ANOVA with Fisher's LSD test. ∗p-value <0.05, ∗∗∗p-value <0.001, ∗∗∗∗p-value <0.0001. Figure S4. a) Blot image quantifying the total amount of secreted proteins from AML12 EDTA-treated and proteinase K treated medium stained with Ponceau S and silver staining. b) Ponceau S staining of a blot image of secreted proteins from conditioned medium from AML12 WT and *Bmal1*-KO cells. c) Protein levels of YWHAH and BMAL1 against the reference protein TUBULIN in WT, KO and LRE mice livers at ZT8 and ZT20. Three biological replicates per genotype were sampled. Statistical comparisons between groups were performed using one-way ANOVA with Fisher's LSD test. ∗pvalue <0.05, ∗∗ p-value <0.01. d) Protein levels of YWHAH and BMAL1 against the reference protein TUBULIN in livers of WT and LKO at ZT8. Western blot: WT (n = 3), LKO (n = 4). A student unpaired t-test was performed to determine statistical differences. ∗∗ p-value <0.01. e) Western blot analyses of extracellular vehicle markers TSG101, CD63 and CD81, and the reference protein Albumin in secreted extracellular vehicles (sEV), soluble secreted proteins and total liver lysate. f) Cryogenic electron microscopy (cryo-EM) images of sEVs from panel f. Scale bars are 20 nm (A) and 50 nm (B,C). g) Dynamic light scattering distribution plot showing the peaks of distinct populations of sEVs that differ in size. h) Western blot analyses showing the presence of YWHAH, the reference protein for extracellular vesicles, CD81, and the nuclear marker Histone H3 for liver samples, respectively and the total protein on gel. i) Western blot analyses of the extracellular vehicle markers TSG101 and CD63 against the reference marker Alix present in conditioned medium from AML12 WT, *Bmal1*-KO and *Ywhah*-KO cells. j) Transmission electron microscopy (TEM) images and k) Nanoparticle Tracking Analysis (NTA) of extracellular vehicles extracted from conditioned medium from AML12 WT (A), *Bmal1*-KO (B) and *Ywhah*-KO cells (C). The scale bars on all images are 500 nm. l) Protein levels of YWHAH, the extracellular vehicle marker Syntenin-1 and the total protein (determined by Zinc staining of the SDS-PAGE gel) present in 4 different fractions extracted from conditioned medium kept on AML12 WT cells for 48 h. Figure S5. a) YWHAH knockout efficiency in AML12 cells. Four biological replicates for each group were sampled. A student unpaired t-test was performed to determine statistical differences. ∗∗ p-value <0.01. b) Trypan blue viability of AML12 WT and *Ywhah*-KO cells. Four biological replicates per genotype were sampled. c) Trypan blue viability of 3T3-L1 WT cells treated with different conditioned media for 6 h. Four biological replicates for each group were sampled. As control, cells were treated with naive medium. Statistical comparisons between groups were performed using two-way ANOVA combined with Fisher's LSD test. ∗p-value <0.05, ∗∗ p-value <0.01. d) Images of 3T3-L1 adipocytes at 10X magnification. The scale bars on all images are 100 μm. Hoescht (Nuclei, blue) and Bodipy (lipid droplet, green) staining of differentiated 3T3L1 adipocytes treated with naive medium (n = 10), conditioned medium from AML12 WT (n = 54) and *Ywhah*-KO cells (n = 48) for 7 days, throughout the differentiation period. The WT conditioned medium group is the same as in Figure 3 (panel c) since these experiments were run in parallel. Number of biological replicates for each conditioned medium treatment is at least eight for each timepoint. Graphs represent mean values and error bars represent standard error of the mean. Statistical comparisons between groups at individual time points were performed using two-way ANOVA with Fisher's LSD test. ∗p-value <0.05, ∗∗ p-value <0.01, ∗∗∗p-value <0.001, ∗∗∗∗p-value <0.0001. e) Oxygen consumption rate of 3T3-L1 adipocytes treated for 6 h with naive medium (n = 8), conditioned medium from AML12 WT (n = 48) or *Ywhah*-KO cells (n = 48). The WT conditioned medium group is the same as in Figure 3 (panel d) since these experiments were run in parallel. Number of biological replicates is eight for each timepoint. Graphs represent mean values and error bars represent standard error of the mean. Statistical comparisons between groups at individual time points were performed using two-way ANOVA with Fisher's LSD test. ∗∗ p-value <0.01. Figure S6. a) Gene ontology enrichment of visceral adipose tissue (VAT) genes positively (red) or negatively (black) correlated with *YWHAH*. b) Age-, sex- and BMIadjusted *YWHAH* correlations to clinical parameters from STARNET cohort (C-reactive protein [CRP], gamma-glutamyl transpeptidase [GGT], alanine transaminase [ALAT], triglycerides [TG], high density lipoprotein [HDL], platelet count [PLT], low density lipoprotein [LDL], aspartate transaminase [ASAT], white blood cells [WBC], haemoglobin [Hgb], non-diseased vessels [ndv] coronary artery disease complexity score [SYNTAX score], diastolic blood pressure [DBP], systolic blood pressure [SBP], coronary artery disease severity score [DUKE], flourosurfactant polymer glycoside [fsp.gly], glycosylated haemoglobin [HbA1c], body mass index [BMI]). Red line denotes statistical significance threshold (–log10(p-value)). c) Network links between CRP and regulatory nodes in GRN6. Figure S1
