# Supplementary material for: The liver clock modulates circadian rhythms in white adipose tissue
Source: Mol Metab. 2025 Sep 12;101:102249. doi: 10.1016/j.molmet.2025.102249 (PMC12489913; doi:10.1016/j.molmet.2025.102249)

Supplementary figure 1.

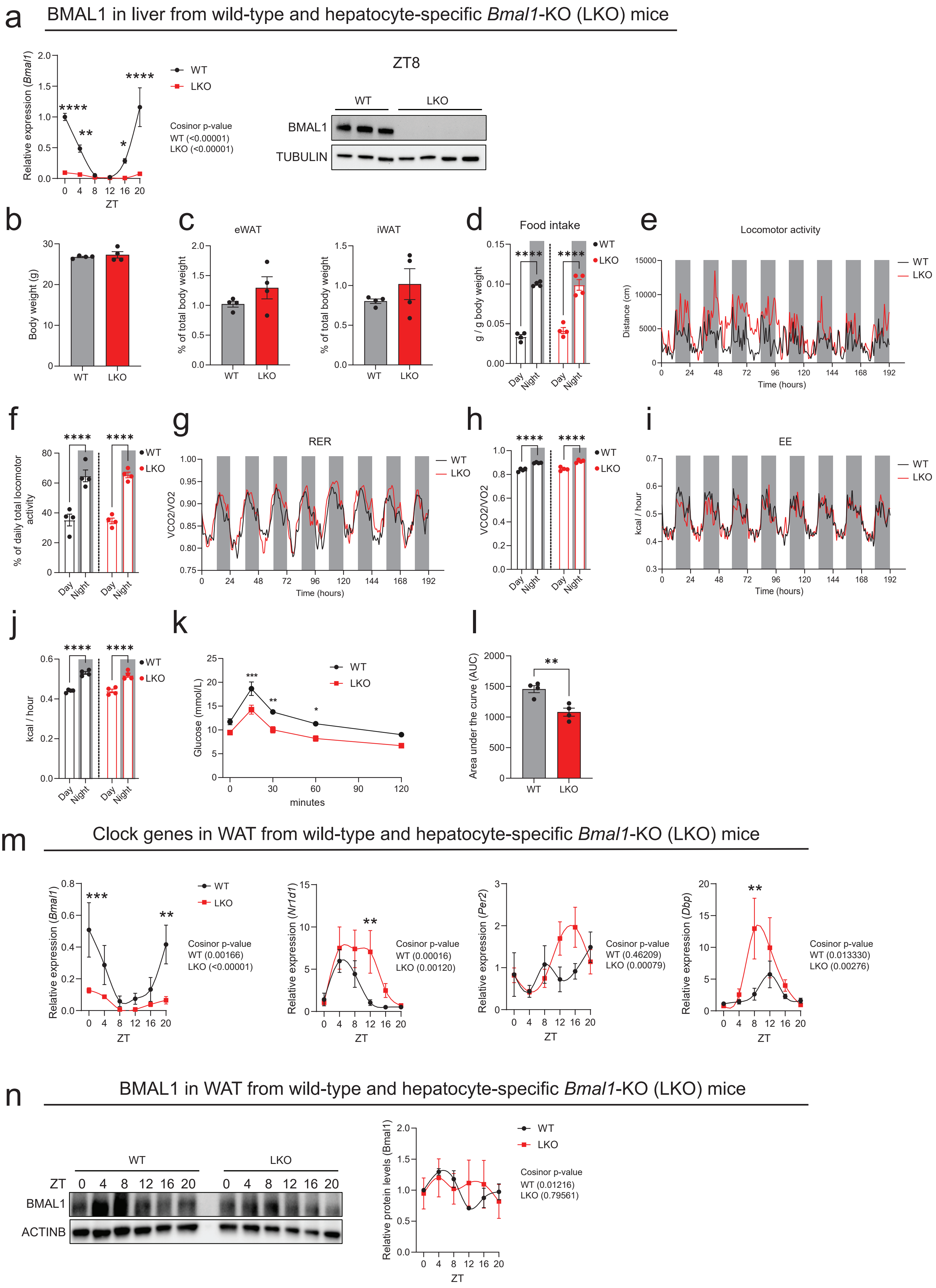

Supplementary figure 2.

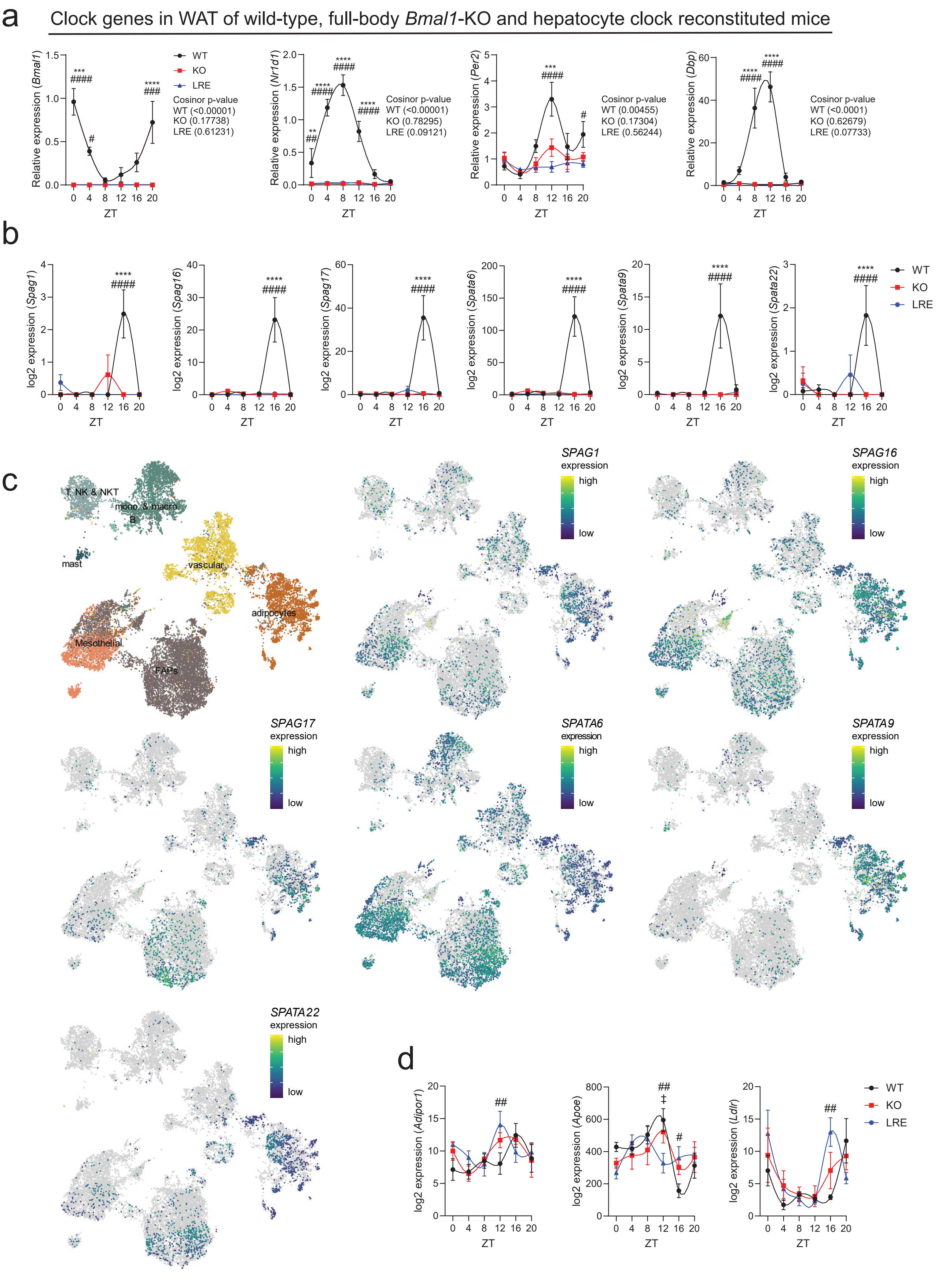

Supplementary figure 3.

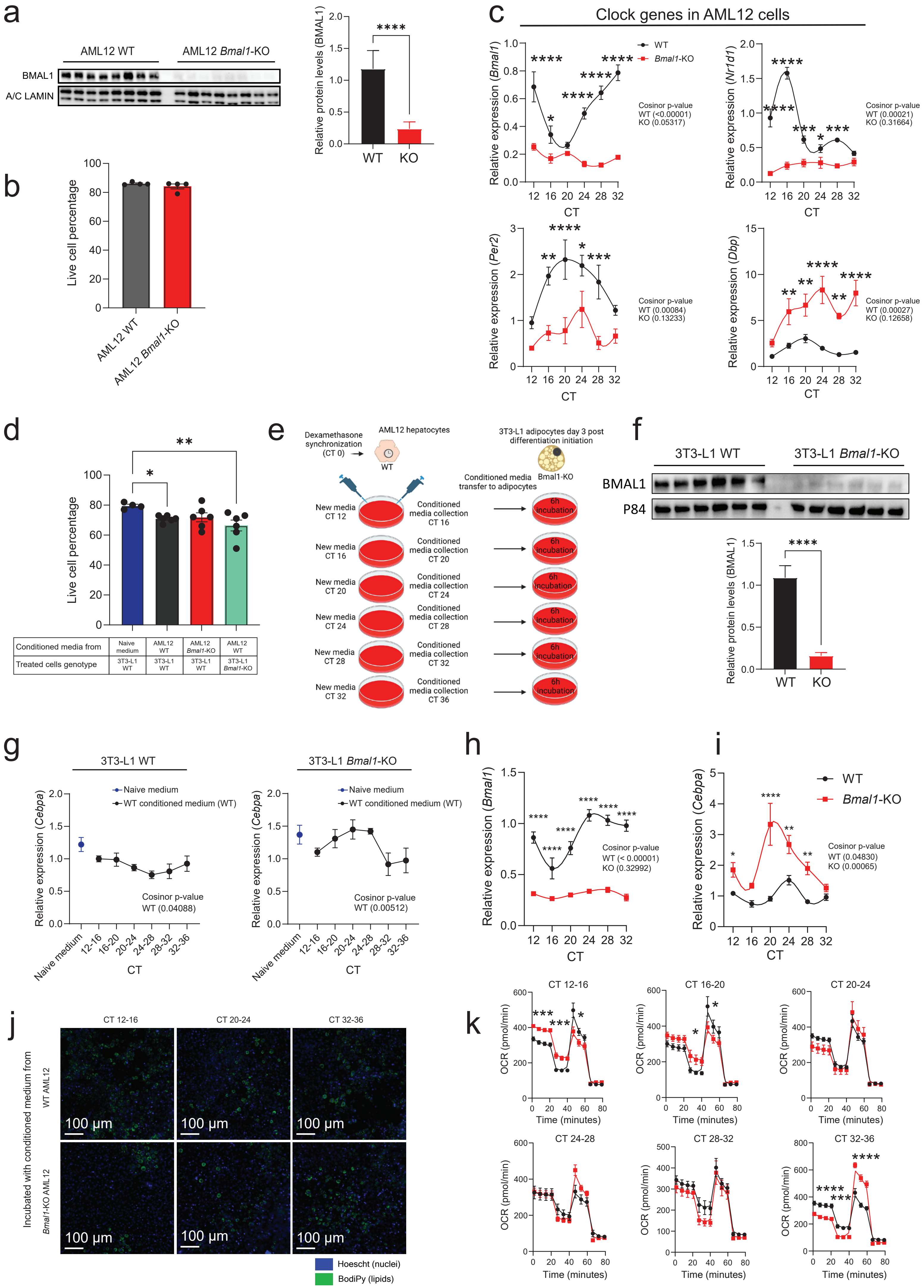

Supplementary figure 4.

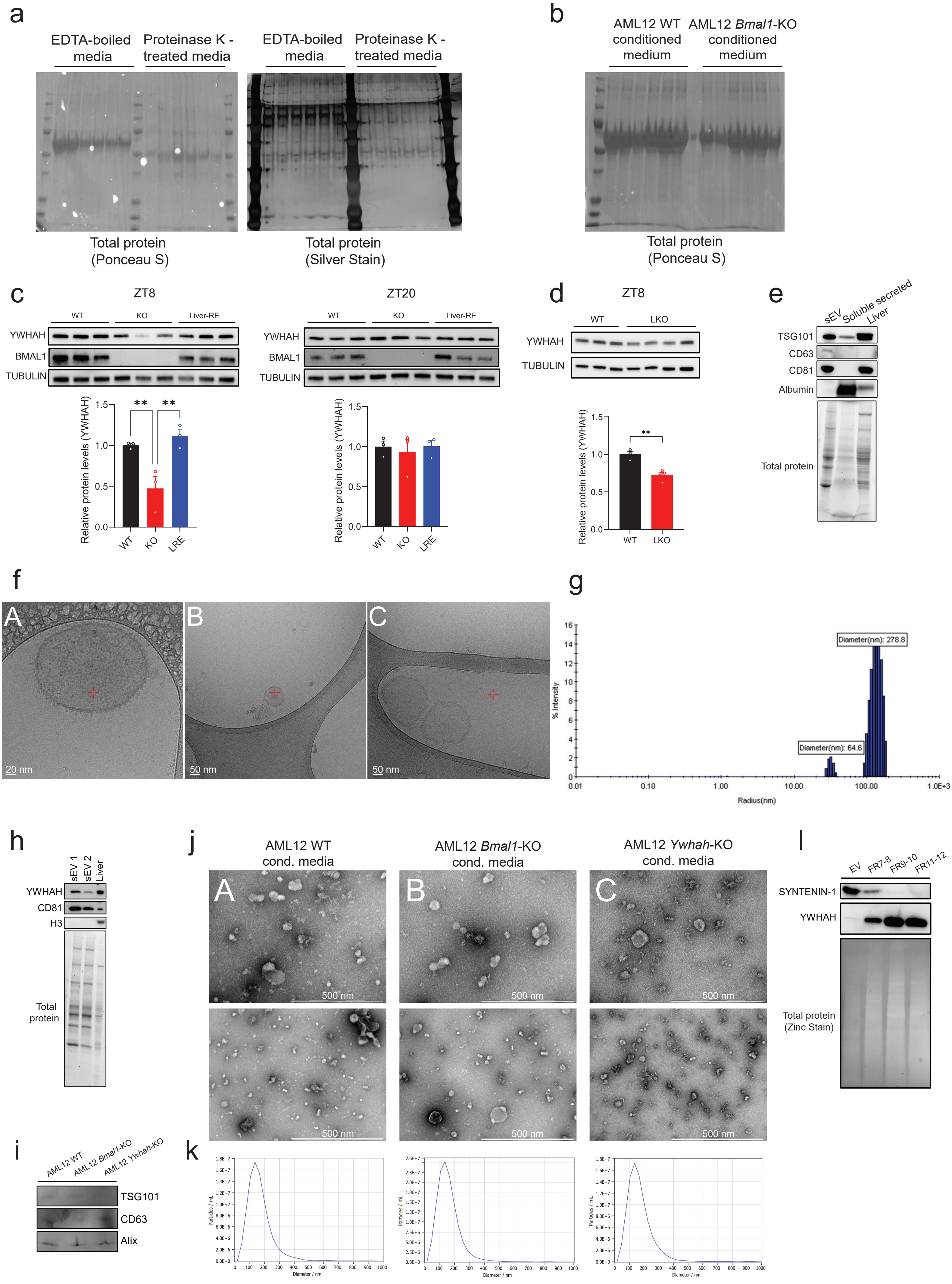

Supplementary figure 5.

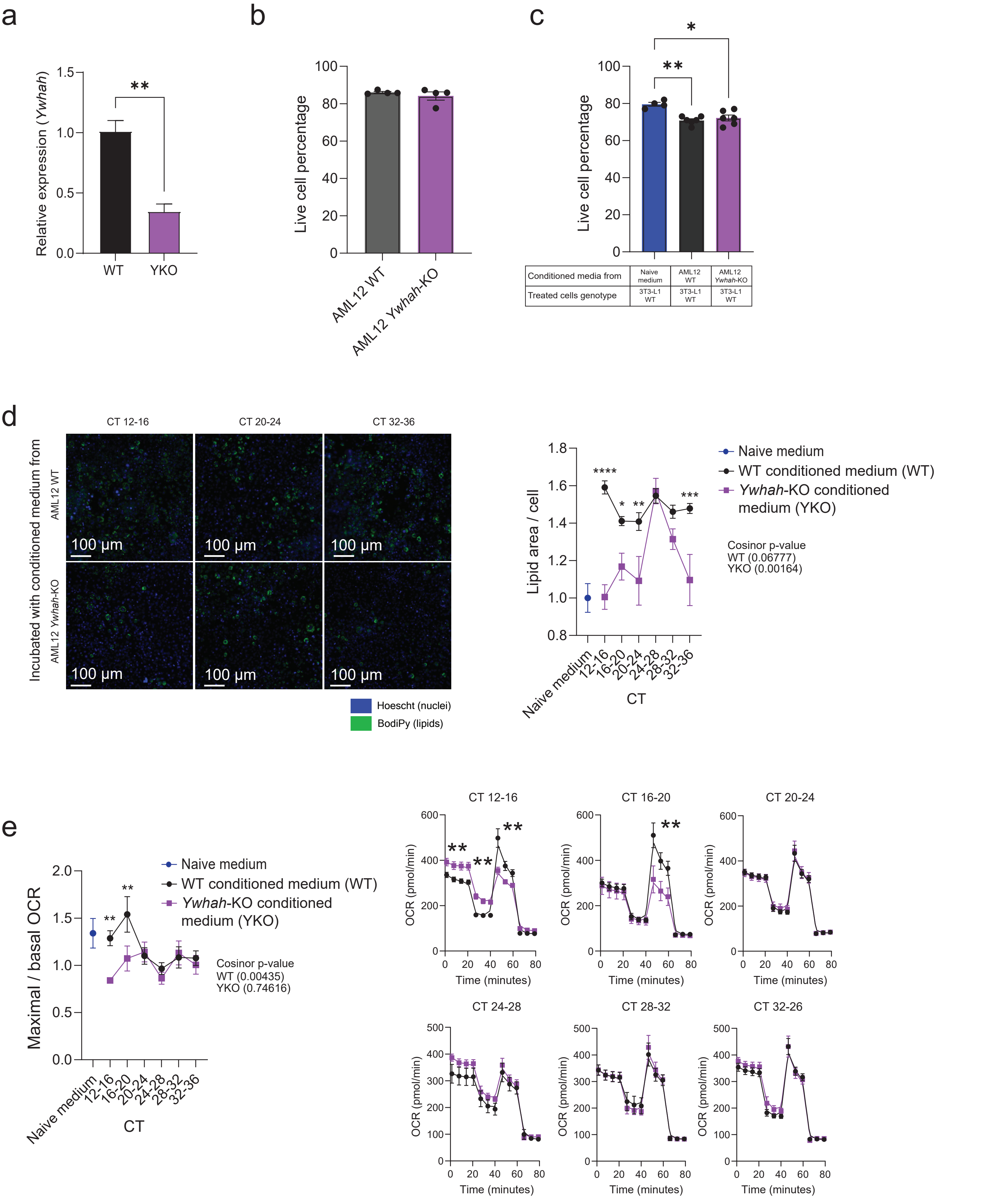

Supplementary figure 6.

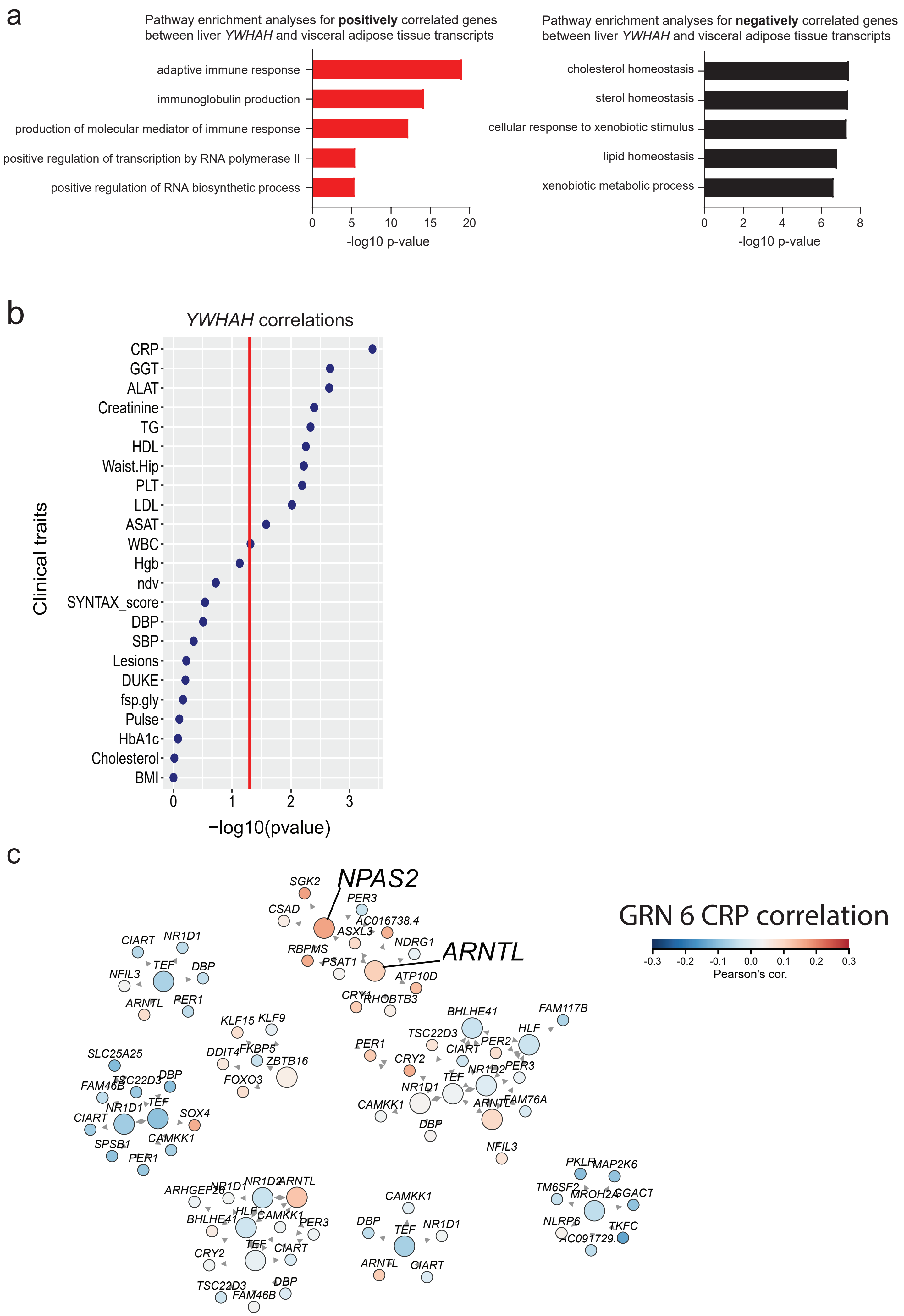

Supplement: Figure S1 — a) Validation of BMAL1 knockdown in liver tissue by qPCR and Western blot. WT (n = 24), LKO (n = 24), four biological replicates per time point. Western blot: WT (n = 3), LKO (n = 4). The graph represents mean values of the relative gene expression and error bars represent standard error of the mean. Cosinor p-values are shown; statistical comparisons between groups at individual time points were performed using two-way ANOVA with Fisher's LSD test. ∗p-value <0.05 ∗∗ p-value <0.01, ∗∗∗∗p-value <0.0001. b-c) Body weight and adipose tissue mass as % of total body weight. Four biological replicates per genotype were sampled. d) 24-hour food intake (light vs dark). The graph represents mean values and error bars represent standard error of the mean, four biological replicates per group. Statistical comparisons between the light and dark period were performed using two-way ANOVA with Fisher's LSD test. ∗∗∗∗p-value <0.0001. e-f) Locomotor activity in light–dark conditions. Four biological replicates per genotype were sampled. Statistical comparisons between the light and dark period were performed using two-way ANOVA with Fisher's LSD test. ∗∗∗∗p-value <0.0001. g-h) Respiratory exchange ratio (RER) in light–dark conditions. Four biological replicates per genotype were sampled. Statistical comparisons between the light and dark period were performed using two-way ANOVA with Fisher's LSD test. ∗∗∗∗p-value <0.0001. i-j) Energy expenditure (EE) measurement in light–dark conditions. Four biological replicates per genotype were sampled. Statistical comparisons between the light and dark period were performed using two-way ANOVA with Fisher's LSD test. ∗∗∗∗p-value <0.0001. k-l) Oral glucose tolerance test curves for WT (black) and LKO (red) mice and comparison of areas under the curve (AUC) between the genotypes. Four biological replicates per genotype were sampled at each time point. Statistical comparisons between groups at individual time points for panel k were performed usi [file mmc6.pdf]
